# Deciphering osteoarthritis genetics across 826,690 individuals from 9 populations

**DOI:** 10.1016/j.cell.2021.07.038

**Published:** 2021-09-02

**Authors:** Cindy G. Boer, Konstantinos Hatzikotoulas, Lorraine Southam, Lilja Stefánsdóttir, Yanfei Zhang, Rodrigo Coutinho de Almeida, Tian T. Wu, Jie Zheng, April Hartley, Maris Teder-Laving, Anne Heidi Skogholt, Chikashi Terao, Eleni Zengini, George Alexiadis, Andrei Barysenka, Gyda Bjornsdottir, Maiken E. Gabrielsen, Arthur Gilly, Thorvaldur Ingvarsson, Marianne B. Johnsen, Helgi Jonsson, Margreet Kloppenburg, Almut Luetge, Sigrun H. Lund, Reedik Mägi, Massimo Mangino, Rob R.G.H.H. Nelissen, Manu Shivakumar, Julia Steinberg, Hiroshi Takuwa, Laurent F. Thomas, Margo Tuerlings, arcOGEN Consortium, John Loughlin, Nigel Arden, Fraser Birrell, Andrew Carr, Panos Deloukas, Michael Doherty, Andrew W. McCaskie, William E.R. Ollier, Ashok Rai, Stuart H. Ralston, Tim D. Spector, Gillian A. Wallis, HUNT All-In Pain, Amy E. Martinsen, Cristen Willer, Egil Andreas Fors, Ingunn Mundal, Knut Hagen, Kristian Bernhard Nilsen, Marie Udnesseter Lie, Sigrid Børte, Ben Brumpton, Jonas Bille Nielsen, Lars G. Fritsche, Wei Zhou, Ingrid Heuch, Kjersti Storheim, ARGO Consortium, Evangelos Tyrpenou, Athanasios Koukakis, Dimitrios Chytas, Dimitrios Stergios Evangelopoulos, Chronopoulos Efstathios, Spiros Pneumaticos, Vasileios S. Nikolaou, Konstantinos Malizos, Lydia Anastasopoulou, Regeneron Genetics Center, Goncalo Abecasis, Aris Baras, Michael Cantor, Giovanni Coppola, Andrew Deubler, Aris Economides, Luca A. Lotta, John D. Overton, Jeffrey G. Reid, Alan Shuldiner, Katia Karalis, Katherine Siminovitch, Christina Beechert, Caitlin Forsythe, Erin D. Fuller, Zhenhua Gu, Michael Lattari, Alexander Lopez, Thomas D. Schleicher, Maria Sotiropoulos Padilla, Louis Widom, Sarah E. Wolf, Manasi Pradhan, Kia Manoochehri, Xiaodong Bai, Suganthi Balasubramanian, Boris Boutkov, Gisu Eom, Lukas Habegger, Alicia Hawes, Olga Krasheninina, Rouel Lanche, Adam J. Mansfield, Evan K. Maxwell, Mona Nafde, Sean O’Keeffe, Max Orelus, Razvan Panea, Tommy Polanco, Ayesha Rasool, William Salerno, Jeffrey C. Staples, Dadong Li, Deepika Sharma, Ilanjana Banerjee, Jonas Bovijn, Adam Locke, Niek Verweij, Mary Haas, George Hindy, Tanima De, Parsa Akbari, Olukayode Sosina, Manuel A.R. Ferreira, Marcus B. Jones, Jason Mighty, Michelle G. LeBlanc, Lyndon J. Mitnaul, George C. Babis, Jason Pui Yin Cheung, Jae Hee Kang, Peter Kraft, Steven A. Lietman, Dino Samartzis, P. Eline Slagboom, Kari Stefansson, Unnur Thorsteinsdottir, Jonathan H. Tobias, André G. Uitterlinden, Bendik Winsvold, John-Anker Zwart, George Davey Smith, Pak Chung Sham, Gudmar Thorleifsson, Tom R. Gaunt, Andrew P. Morris, Ana M. Valdes, Aspasia Tsezou, Kathryn S.E. Cheah, Shiro Ikegawa, Kristian Hveem, Tõnu Esko, J. Mark Wilkinson, Ingrid Meulenbelt, Ming Ta Michael Lee, Joyce B.J. van Meurs, Unnur Styrkársdóttir, Eleftheria Zeggini

**Affiliations:** 1 Department of Internal Medicine, Erasmus MC, Medical Center, 3015CN Rotterdam, the Netherlands; 2 Institute of Translational Genomics, Helmholtz Zentrum München, German Research Center for Environmental Health, 85764 Neuherberg, Germany; 3 deCODE Genetics/Amgen Inc., 102 Reykjavik, Iceland; 4 Genomic Medicine Institute, Geisinger Health System, Danville, PA 17822, USA; 5 Department of Biomedical Data Sciences, Section Molecular Epidemiology, Postzone S05-P Leiden University Medical Center, 2333ZC Leiden, the Netherlands; 6 Department of Psychiatry, Li Ka Shing Faculty of Medicine, The University of Hong Kong, Pokfulam, Hong Kong, China; 7 MRC Integrative Epidemiology Unit (IEU), Bristol Medical School, University of Bristol, Oakfield House, Oakfield Grove, Bristol BS8 2BN, UK; 8 Musculoskeletal Research Unit, Translation Health Sciences, Bristol Medical School, University of Bristol, Southmead Hospital, Bristol BS10 5NB, UK; 9 Estonian Genome Center, Institute of Genomics, University of Tartu, 51010 Tartu, Estonia; 10 K.G. Jebsen Center for Genetic Epidemiology, Department of Public Health and Nursing, Faculty of Medicine and Health Sciences, Norwegian University of Science and Technology, 7491 Trondheim, Norway; 11 Laboratory for Statistical and Translational Genetics, RIKEN Center for Integrative Medical Sciences, Kanagawa 230-0045, Japan; 12 4^th^ Psychiatric Department, Dromokaiteio Psychiatric Hospital, 12461 Athens, Greece; 13 1^st^ Department of Orthopaedics, KAT General Hospital, 14561 Athens, Greece; 14 Faculty of Medicine, University of Iceland, 101 Reykjavik, Iceland; 15 Department of Orthopedic Surgery, Akureyri Hospital, 600 Akureyri, Iceland; 16 Institute of Clinical Medicine, Faculty of Medicine, University of Oslo, 0316 Oslo, Norway; 17 Research and Communication Unit for Musculoskeletal Health (FORMI), Department of Research, Innovation and Education, Division of Clinical Neuroscience, Oslo University Hospital, 0424 Oslo, Norway; 18 Department of Medicine, Landspitali The National University Hospital of Iceland, 108 Reykjavik, Iceland; 19 Departments of Rheumatology and Clinical Epidemiology, Leiden University Medical Center, 9600, 23OORC Leiden, the Netherlands; 20 Department of Twin Research and Genetic Epidemiology, Kings College London, London SE1 7EH, UK; 21 Department of Orthopaedics, Leiden University Medical Center, 9600, 23OORC Leiden, the Netherlands; 22 Department of Biostatistics, Epidemiology and Informatics, Perelman School of Medicine, University of Pennsylvania, Philadelphia, PA 19104, USA; 23 Daffodil Centre, The University of Sydney, a joint venture with Cancer Council NSW, Sydney, NSW 1340, Australia; 24 Laboratory for Bone and Joint Diseases, RIKEN Center for Integrative Medical Sciences, Tokyo 108-8639, Japan; 25 Department of Orthopedic Surgery, Shimane University, Shimane 693-8501, Japan; 26 Department of Clinical and Molecular Medicine, Norwegian University of Science and Technology, 7491 Trondheim, Norway; 27 BioCore-Bioinformatics Core Facility, Norwegian University of Science and Technology, 7491 Trondheim, Norway; 28 Clinic of Laboratory Medicine, St. Olavs Hospital, Trondheim University Hospital, 7030 Trondheim, Norway; 29 2^nd^ Department of Orthopaedics, National and Kapodistrian University of Athens, Medical School, Nea Ionia General Hospital Konstantopouleio, 14233 Athens, Greece; 30 Department of Orthopaedics and Traumatology, The University of Hong Kong, Pokfulam, Hong Kong, China; 31 Department of Medicine, Brigham and Women’s Hospital, 181 Longwood Ave, Boston, MA 02115, USA; 32 Department of Epidemiology, Harvard T.H. Chan School of Public Health, 677 Huntington Avenue, Boston, MA 02115, USA; 33 Musculoskeletal Institute, Geisinger Health System, Danville, PA 17822, USA; 34 Department of Orthopaedic Surgery, Rush University Medical Center, Chicago, IL 60612, USA; 35 Department of Research, Innovation and Education, Division of Clinical Neuroscience, Oslo University Hospital and University of Oslo, 0450 Oslo, Norway; 36 Department of Neurology, Oslo University Hospital, 0424 Oslo, Norway; 37 Population Health Sciences, Bristol Medical School, University of Bristol, Bristol BS8 2BN, UK; 38 Li Ka Shing Faculty of Medicine, The University of Hong Kong, Pokfulam, Hong Kong, China; 39 Centre for Genetics and Genomics Versus Arthritis, Centre for Musculoskeletal Research, University of Manchester, Manchester M13 9LJ, UK; 40 Faculty of Medicine and Health Sciences, School of Medicine, University of Nottingham, Nottingham, Nottinghamshire NG5 1PB, UK; 41 Laboratory of Cytogenetics and Molecular Genetics, Faculty of Medicine, University of Thessaly, Larissa 411 10, Greece; 42 School of Biomedical Sciences, The University of Hong Kong, Pokfulam, Hong Kong, China; 43 HUNT Research Center, Department of Public Health and Nursing, Faculty of Medicine and Health Sciences, Norwegian University of Science and Technology, 7600 Levanger, Norway; 44 Department of Oncology and Metabolism and Healthy Lifespan Institute, University of Sheffield, Sheffield S10 2RX, UK; 45 Institute of Biomedical Sciences, Academia Sinica, 115 Taipei, Taiwan; 46 TUM School of Medicine, Technical University of Munich and Klinikum Rechts der Isar, 81675 Munich, Germany

**Keywords:** osteoarthritis, genome-wide association meta-analysis, genetic architecture, functional genomics, effector genes, drug targets

## Abstract

Osteoarthritis affects over 300 million people worldwide. Here, we conduct a genome-wide association study meta-analysis across 826,690 individuals (177,517 with osteoarthritis) and identify 100 independently associated risk variants across 11 osteoarthritis phenotypes, 52 of which have not been associated with the disease before. We report thumb and spine osteoarthritis risk variants and identify differences in genetic effects between weight-bearing and non-weight-bearing joints. We identify sex-specific and early age-at-onset osteoarthritis risk loci. We integrate functional genomics data from primary patient tissues (including articular cartilage, subchondral bone, and osteophytic cartilage) and identify high-confidence effector genes. We provide evidence for genetic correlation with phenotypes related to pain, the main disease symptom, and identify likely causal genes linked to neuronal processes. Our results provide insights into key molecular players in disease processes and highlight attractive drug targets to accelerate translation.

## Highlights

• A multicohort study identifies 52 previously unknown osteoarthritis genetic risk variants
• Similarities and differences in osteoarthritis genetic risk depend on joint sites
• Osteoarthritis genetic components are associated with pain-related phenotypes
• High-confidence effector genes highlight potential targets for drug intervention

## Graphical abstract

**Figure:**
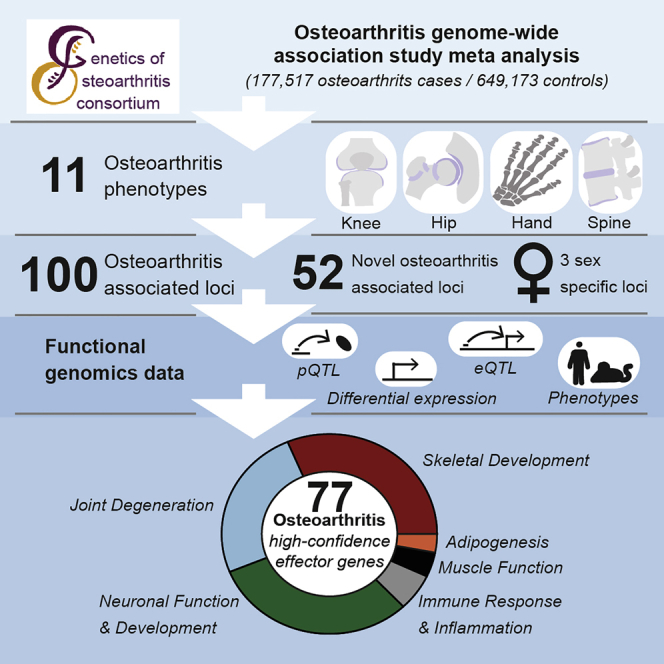

## Teaser

A multicohort genome-wide association meta-analysis of osteoarthritis highlights the impact of joint site types on the features of genetic risk variants and the link between osteoarthritis genetics and pain-related phenotypes, pointing toward potential targets for therapeutic intervention.

## Introduction

Osteoarthritis is one of the leading causes of disability and pain worldwide, with over 300 million people affected (GBD 2017 Disease and Injury Incidence and Prevalence Collaborators, 2018). Currently no curative treatments are available, and management strategies focus on symptom alleviation through pain relief and arthroplasty. A detailed understanding of disease etiopathology and new drug targets are therefore urgently needed.

Osteoarthritis is a complex degenerative disease of the whole joint, characterized by cartilage degeneration, subchondral bone thickening, osteophyte formation, synovial inflammation, and structural alterations of the joint capsule, ligaments, and associated muscles (Hunter and Bierma-Zeinstra, 2019). Recently, advances were made in elucidating the genetic background of osteoarthritis, using genome-wide association studies (GWAS) (Styrkarsdottir et al., 2018; Tachmazidou et al., 2019; Tachmazidou et al., 2017; Zengini et al., 2018), with 96 statistically independent risk variants reported to date. These variants only explain a small proportion of the phenotypic variance (Tachmazidou et al., 2019) and are mainly associated with osteoarthritis affecting the knee and hip joints.

Osteoarthritis can affect every synovial joint and an increase in body mass index (BMI) is associated with risk of disease (Geusens and van den Bergh, 2016). A better understanding of the genetic differences between weight bearing (knee, hip, and spine) and non-weight bearing joints (hand, finger, and thumb) is needed to help disentangle the metabolic and biomechanical effects contributing to disease development. Here, we conducted a GWAS meta-analysis across knee, hip, finger, thumb, and spine osteoarthritis phenotypes in 826,690 individuals of European and East Asian descent. We integrated functional genomics analyses from disease-relevant tissue, including gene expression, protein abundance and genome-wide methylation, mouse knockout model and monogenic human disease phenotyping data, and complementary computational fine-mapping, colocalization, and causal inference approaches to identify likely effector genes and facilitate much-needed translation into therapies by enhancing our understanding of disease etiopathology.

## Results

### Genetic architecture

#### Identification of osteoarthritis risk variants

**Figure 1:**
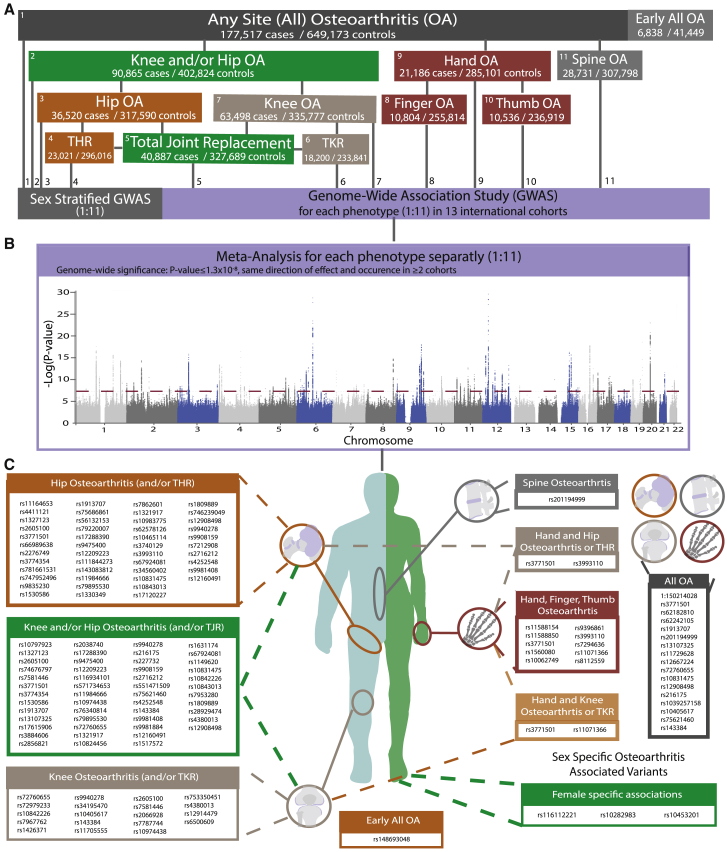
Genetic architecture Graphical summary of the Genetics of Osteoarthritis Consortium workflow and results. (A) Overview of the 11 defined osteoarthritis phenotypes, sex specific analysis, their relationship with each other and their sample sizes (cases/controls). TKR, total knee replacement; THR, total hip replacement. (B) Merged Manhattan-plot of all individual meta-analysis results of all 11 examined osteoarthritis phenotypes. The dashed line represents the genome-wide significance threshold p = 1.3 × 10^−8^. (C) Graphical overview of all lead genome-wide significant independent osteoarthritis associated single nucleotide variants (SNVs) and the osteoarthritis phenotypes with which they are associated. See also Table S1.

We performed GWAS meta-analyses for osteoarthritis across 13 international cohorts stemming from 9 populations (Table S1), in up to 826,690 individuals (177,517 osteoarthritis patients). This is a substantial (2.3-fold) increase of osteoarthritis patient numbers compared to the largest osteoarthritis GWAS to date. Two of the cohorts are of East Asian and 11 of the cohorts are of European descent. We defined 11 phenotypes encompassing all major sites for osteoarthritis (Figure 1; Table S1; STAR Methods). We found 11,897 genome-wide significantly associated single nucleotide variants (SNVs) using a threshold of p < 1.3 × 10^−8^, to account for the effective number of independent tests. We applied conditional analyses within phenotype and identified 223 independent associations, some of which overlap across phenotypes (Figure 1; Table 1). Eighty-four variants have not been associated with osteoarthritis before. We investigated the previously reported osteoarthritis-loci and found that 87 out of 96 replicated in the same direction at nominal significance (Table S2).

**Table 1:** Summary results for all genome-wide significant osteoarthritis associated SNVs

Genome-wide association study	Cases/controls	Signals^b^	New signals^b^	Known signals^b^
All osteoarthritis^a^	177,517/649,173	21	8	13
Knee and/or hip osteoarthritis	89,741/400,604	31	9	22
Hip osteoarthritis	36,445/316,943	45	17	28
Knee osteoarthritis	62,497/333,557	24	11	13
Total hip replacement	23,021/296,016	38	12	26
Total knee replacement	18,200/233,841	10	4	6
Total joint replacement	40,887/327,689	37	12	25
Hand osteoarthritis	20,901/282,881	7	5	2
Finger osteoarthritis	10,804/255,814	5	3	2
Thumb osteoarthritis	10,536/236,919	4	2	2
Spine osteoarthritis	28,3721/3057,578	1	1	0
Total		223	84	139
Total independent signals across phenotypes^c^		100	52	48

**Sex-specific analysis**				

Female total hip replacement	11,089/67,516	2	2	0
Female all osteoarthritis	90,838/192,697	1	1	0

**Early-onset osteoarthritis analysis**				

Early all osteoarthritis	6,838/41,449	1	1	0

Signals reported here are genome-wide significant (p < 1.3 × 10^−8^) with the exception of the early-onset analysis (p < 5 × 10^−8^).

a Cases are any-site osteoarthritis: hip, knee, hand, finger, thumb, and spine.

b Signals numbers are based per defined osteoarthritis phenotype, new/known are based on previously reported osteoarthritis loci.

c Independence calculated within and across osteoarthritis phenotypes, the lead SNV is assigned to the most significant phenotype (Table S3).

We used conditional analyses to identify associations that do not overlap across disease phenotype definitions. We identified 100 unique and independent variant associations, 60 of which were associated with more than one osteoarthritis phenotype. Fifty-two variants have not been associated with any osteoarthritis phenotype before (Tables 2 and S3). For each of the 100 association signals, we defined the lead SNV as the risk variant with the strongest statistical evidence for association. Six lead SNVs are coding (all missense), 59 reside within a gene transcript, and 35 are intergenic.

**Table 2:** Summary statistics of the 100 independent genome-wide significant SNVs

Osteoarthritis phenotype	Other osteoarthritis phenotypes	SNV	Chr:pos	EA	NEA	EAF	OR	95% CI	p	Annotation	Nearest gene	WtGrp
**New SNVs**												

FingerOA		rs11588154	1:55301936	T	G	0.17	0.83	0.79–0.88	6.08 × 10^−10^	intron	*C1orf177*	2
HipOA	THR	rs4411121	1:118757034	T	C	0.31	1.07	1.05–1.09	2.16 × 10^−11^	intergenic	*SPAG17*	0
THR	HipOA, TJR	rs1327123	1:184014593	C	G	0.35	0.91	0.89–0.93	2.44 × 10^−16^	intergenic	*TSEN15*	0
ThumbOA		rs11588850	1:227927242	A	G	0.82	0.87	0.84–0.91	3.53 × 10^−10^	intron	*SNAP47*	2
KneeHipOA	KneeOA	rs74676797	2:633063	A	G	0.82	1.05	1.03–1.07	6.39 × 10^−10^	intergenic	*TMEM18*	0
THR	HipOA	rs66989638	2:106689736	A	G	0.13	1.12	1.08–1.15	3.31 × 10^−11^	intron	*C2orf40*	1
THR		rs2276749	3:11643465	T	C	0.05	0.86	0.82–0.90	3.34 × 10^−9^	missense p.Ile37Met	*VGLL4*	1
AllOA		rs62242105	3:20630395	A	G	0.33	0.97	0.96–0.98	2.93 × 10^−9^	intergenic	*RNU6-815P*	NA
HipOA		rs781661531	3:187051013	T	C	7 × 10^−4^	0.11	0.05-0.21	8.36 × 10^−11^	intergenic	*RTP4*	NA
HipOA		rs747952496	3:188311659	A	G	4 × 10^−4^	7.02	3.93–12.55	4.91 × 10^−11^	intron	*LPP*	NA
HipOA		rs9835230	3:189735461	A	G	0.24	1.07	1.04–1.09	1.34 × 10^−9^	intron	*LEPREL1*	1
AllOA	SpineOA	rs201194999	4:66666895	T	C	0.3	0.88	0.85–0.92	3.05 × 10^−9^	intergenic	*RNU2-40P*	1
AllOA		rs11729628	4:121584282	T	G	0.24	0.97	0.96–0.98	4.74 × 10^−9^	intergenic	*RP11-501E14.1*	NA
THR		rs75686861	4:145621328	A	G	0.09	1.12	1.08–1.16	3.04 × 10^−9^	intron	*HHIP*	0
KneeOA		rs2066928	5:30843787	A	G	0.48	0.96	0.95–0.97	1.20 × 10^−8^	intergenic	*RPL19P11*	1
THR	HipOA	rs56132153	5:67825133	A	C	0.61	1.07	1.05–1.09	3.80 × 10^−9^	intron	*CTC-537E7.1*	0
HandOA		rs1560080	5:115338732	A	G	0.83	0.91	0.88–0.94	9.61 × 10^−9^	intron	*AQPEP*	1
KneeHipOA	TJR, AllOA, HipOA, THR	rs17615906	5:128018413	T	C	0.84	0.95	0.93–0.96	3.76 × 10^−11^	intron	*SLC27A6*	1
HandOA	ThumbOA, KneeOA	rs10062749	5:141805088	T	G	0.27	1.08	1.06–1.11	2.04 × 10^−09^	intron	*AC005592.2*	1
FingerOA	HandOA	rs9396861	6:18404133	A	C	0.61	1.13	1.09–1.17	9.35 × 10^−11^	intron	*RNF144B*	2
TJR		rs2038740	6:35114542	T	C	0.72	0.94	0.93–0.96	6.20 × 10^−10^	intron	*TCP11*	1
TJR		rs116934101	7:101775597	A	G	0.27	1.106	1.04–1.08	7.12 × 10^−9^	intron	*CUX1*	1
AllOA		rs12667224	7:114024316	A	G	0.52	0.97	0.96–0.98	1.66 × 10^−9^	intron	*FOXP2*	NA
KneeHipOA		rs571734653	7:137143697	A	C	3 × 10^−4^	6.03	3.30–11.03	5.56 × 10^−9^	intron	*DGKI*	NA
TKR		rs7787744	7:150521096	A	G	0.67	1.08	1.05–1.11	1.29 × 10^−9^	upstream_gene	*AOC1*	0
TJR		rs76340814	9:98321412	A	G	0.05	0.89	0.86–0.92	1.87 × 10^−9^	intergenic	*RP11-332M4.1*	0
THR	HipOA, TJR, KneeHipOA	rs79895530	9:110416422	T	C	0.13	0.88	0.85–0.91	3.86 × 10^−14^	intergenic	*RNU6-996P*	0
HipOA		rs7862601	9:118343026	A	G	0.62	0.94	0.92–0.96	6.19 × 10^−9^	intergenic	*RP11-284G10.1*	0
HipOA		rs10983775	9:120521100	T	C	0.54	0.95	0.93–0.97	4.65 × 10^−9^	intergenic	*RP11-281A20.2*	0
HipOA		rs10465114	9:129917824	A	G	0.22	1.06	1.04–1.09	9.04 × 10^−9^	intron	*RALGPS1*	0
THR	HipOA	rs3740129	10:73767859	A	G	0.46	1.08	1.05–1.10	1.70 × 10^−11^	Missense p.Arg357Gln	*CHST3*	0
TJR		rs10824456	10:78615458	C	G	0.58	10.95	0.94–0.97	1.16 × 10^−8^	intergenic	*KCNMA1*	1
HandOA	THR	rs3993110	11:12794530	A	C	0.61	1.09	1.06–1.11	3.75 × 10^−11^	intron	*TEAD1*	1
KneeHipOA		rs1631174	11:47974373	A	C	0.34	1.04	1.03–1.05	7.28 × 10^−9^	regulatory_region	*PTPRJ*	1
TKR	KneeOA	rs72979233	11:74355523	A	G	0.75	0.92	0.89–0.95	2.52 × 10^−9^	intron	*POLD3*	1
TJR	AllOA, KneeHipOA, HipOA, THR	rs10831475	11:95796907	A	G	0.81	1.08	1.05–1.10	5.89 × 10^−12^	intron	*MAML2*	1
KneeHipOA	KneeOA, TKR	rs10842226	12:23959589	A	G	0.42	1.04	1.03–1.06	4.68 × 10^−10^	intron	*SOX5*	1
TKR	KneeOA	rs7967762	12:48420214	T	C	0.16	1.11	1.07–1.15	4.41 × 10^−10^	upstream_gene	*RP1-228P16.4*	1
KneeOA		rs1426371	12:108629780	A	G	0.27	10.95	0.93–0.97	8.86 × 10^−10^	intron	*WSCD2*	0
KneeOA		rs58973023	13:42959133	A	T	0.49	1.06	1.04–1.08	4.72 × 10^−10^	intergenic	*FABP3P2*	1
TJR		rs28929474	14:94844947	T	C	0.02	0.81	0.76–0.86	1.06 × 10^−10^	Missense p.Glu366Gln	*SERPINA1*	0
THR	HipOA	rs746239049	15:63067433	D	I	0.21	0.90	0.87–0.93	8.19 × 10^−12^	intron	*TLN2*	0
KneeOA		rs12914479	15:99174828	C	G	0.66	1.04	1.03–1.06	7.12 × 10^−9^	intergenic	*RP11-35O15.1*	0
KneeOA		rs6500609	16:4515334	C	G	0.11	0.94	0.91–0.96	5.16 × 10^−9^	intron	*NMRAL1*	1
TJR		rs227732	17:54769890	T	C	0.3	1.06	1.04–1.09	1.61 × 10^−9^	intergenic	*NOG*	0
KneeHipOA	HipOA, AllOA	rs9908159	17:54841961	T	C	0.51	1.04	1.03–1.05	4.44 × 10^−11^	intergenic	*C17orf67*	1
AllOA		rs1039257158	18:77950448	T	C	6 × 10^−4^	3.62	2.35–5.60	6.56 × 10^−9^	intron	*PARD6G*	NA
KneeHipOA		rs551471509	19:9943264	T	C	6 × 10^−4^	0.18	0.10–0.32	1.15 × 10^−8^	upstream_gene	*CTD-2623N2.11*	NA
HandOA	FingerOA	rs8112559	19:46390455	C	G	0.89	1.13	1.09–1.18	7.32 × 10^−11^	upstream_gene	*IRF2BP1*	2
TJR		rs9981884	21:40585633	A	G	0.49	0.95	0.94–0.97	7.93 × 10^−9^	intron	*BRWD1*	1
KneeOA		rs11705555	22:28206912	A	C	0.76	1.05	1.03–1.07	3.00 × 10^−9^	regulatory_region	*MN1*	1
THR	TJR, HipOA	rs12160491	22:38195796	A	G	0.71	0.93	0.90–0.95	1.28 × 10^−10^	intergenic	*H1F0*	0

**Previously reported**												

HipOA	THR, TJR, AllOA, KneeHipOA	rs11164653	1:103464210	T	C	0.41	0.92	0.91–0.94	2.77 × 10^−18^	intron	*COL11A1*	1
AllOA		1:150214028	1:150214028	D	I	0.38	1.04	1.02–1.05	8.58 × 10^−10^	intergenic	*RNU2-17P*	NA
TJR		rs10797923	1:183901966	T	C	0.69	1.05	1.04–1.07	6.20 × 10^−9^	intron	*COLGALT2*	0
TJR	KneeHipOA, KneeOA, HipOA, THR	rs2605100	1:219644224	A	G	0.32	1.07	1.05–1.09	4.49 × 10^−15^	intergenic	*RP11-95P13.1*	1
KneeHipOA	KneeOA	rs7581446	2:33423801	T	C	0.48	0.95	0.94–0.97	4.87 × 10^−11^	intron	*LTBP1*	1
AllOA	HipOA, TJR, THR, ThumbOA, KneeHipOA, HandOA	rs3771501	2:70717653	A	G	0.47	1.04	1.03–1.05	4.05 × 10^−15^	intron	*TGFA*	NA
AllOA		rs62182810	2:204387482	A	G	0.54	1.03	1.02–1.04	3.82 × 10^−9^	intron	*RAPH1*	NA
THR	KneeHipOA, TJR, HipOA	rs3774354	3:52817675	A	G	0.37	1.10	1.07–1.12	1.40 × 10^−16^	intron	*ITIH1*	0
TJR	TKR, HipOA, AllOA, KneeOA, THR, KneeHipOA	rs1530586	4:1760927	T	C	0.8	1.09	1.06–1.11	3.34 × 10^−14^	regulatory_region	*TACC3*	0
THR	TJR, HipOA, KneeHipOA, AllOA	rs1913707	4:13039440	A	G	0.6	1.09	1.06–1.11	1.23 × 10^−13^	intergenic	*RNU6-962P*	1
AllOA	HipOA, KneeHipOA	rs13107325	4:103188709	T	C	0.07	1.08	1.06–1.10	3.25 × 10^−17^	missense p.Ala391Thr	*SLC39A8*	0
KneeHipOA	HipOA	rs3884606	5:170871074	A	G	0.52	0.96	0.95–0.97	8.96 × 10^−10^	intron	*FGF18*	1
HipOA		rs79220007	6:26098474	T	C	0.93	0.90	0.87–0.93	2.22 × 10^−9^	3_prime_UTR	*HFE*	0
KneeHipOA		rs2856821	6:33046742	T	C	0.79	1.11	1.03–1.06	5.71 × 10^−9^	intron	*HLA-DPA1*	0
THR	KneeHipOA, HipOA, TJR	rs17288390	6:45384018	T	C	0.65	0.92	0.90–0.94	9.16 × 10^−13^	intron	*RUNX2*	0
THR	HipOA, TJR	rs9475400	6:55638258	T	C	0.1	1.15	1.10–1.19	1.73 × 10^−13^	intron	*BMP5*	0
THR	HipOA, TJR	rs12209223	6:76164589	A	C	0.11	1.22	1.18–1.26	1.92 × 10^−29^	intron	*FILIP1*	1
HipOA	THR	rs111844273	7:18436337	A	G	0.02	1.26	1.18–1.34	1.05 × 10^−12^	intron	*HDAC9*	0
THR	HipOA	rs143083812	7:128843410	T	C	1.1 × 10^−3^	3.30	2.34–4.66	1.11 × 10^−11^	missense p.Arg173Cys	*SMO*	NA
THR	HipOA, TJR	rs11984666	8:130730280	A	C	0.2	0.90	0.87–0.92	1.69 × 10^−15^	intergenic	*RP11-274M4.1*	0
KneeHipOA	KneeOA	rs10974438	9:4291928	A	C	0.65	1.04	1.03–1.06	7.39 × 10^−11^	intron	*GLIS3*	1
KneeHipOA	TKR, KneeOA, TJR, AllOA	rs72760655	9:116916214	A	C	0.33	1.05	1.03–1.06	5.97 × 10^−13^	upstream_gene	*COL27A1*	1
THR	HipOA	rs1330349	9:117840742	C	G	0.59	1.10	1.07–1.12	6.47 × 10^−17^	intron	*TNC*	0
THR	HipOA, TJR	rs1321917	9:119324929	C	G	0.41	1.10	1.08–1.13	9.87 × 10^−19^	intron	*ASTN2*	1
THR	HipOA	rs62578126	9:129375338	T	C	0.37	0.92	0.90–0.94	1.39 × 10^−12^	intron	*RP11-123K19.1*	0
KneeHipOA	TJR	rs1517572	11:28829882	A	C	0.41	1.04	1.03–1.05	6.79 × 10^−10^	intron	*RP11-115J23.1*	1
THR	HipOA, TJR	rs67924081	11:65342981	A	G	0.74	1.10	1.07–1.12	2.14 × 10^−13^	upstream_gene	*EHBP1L1*	1
THR	HipOA	rs34560402	11:66872320	T	C	0.06	0.86	0.82–0.90	2.64 × 10^−10^	intergenic	*KDM2A*	0
KneeHipOA		rs1149620	11:76506572	A	T	0.44	0.96	0.95–0.97	2.87 × 10^−9^	intron	*TSKU*	1
FingerOA		rs7294636	12:15054016	A	G	0.37	1.16	1.12–1.20	2.99 × 10^−16^	intron	*C12orf60*	1
THR	TJR, KneeHipOA, HipOA	rs10843013	12:28025196	A	C	0.78	0.86	0.84–0.88	2.53 × 10^−30^	intergenic	*RP11-993B23.1*	0
THR	HipOA	rs17120227	12:59289349	T	C	0.07	1.17	1.12–1.22	7.21 × 10^−13^	intron	*LRIG3*	0
KneeHipOA	TJR	rs7953280	12:94136009	C	G	0.5	1.04	1.03–1.06	4.84 × 10^−12^	intron	*CRADD*	0
KneeOA		rs753350451	12:123732769	D	I	0.2	0.93	0.91–0.95	3.36 × 10^−10^	intron	*C12orf65*	0
TJR	HipOA, THR	rs1809889	12:124801226	T	C	0.28	1.07	1.05–1.09	5.70 × 10^−14^	downstream_gene	*FAM101A*	0
KneeOA	KneeHipOA	rs4380013	15:50759428	A	G	0.19	1.06	1.04–1.08	8.73 × 10^−10^	intron	*USP8*	1
HandOA	KneeOA, TKR, FingerOA, ThumbOA	rs11071366	15:58334244	A	T	0.61	0.90	0.88–0.92	4.88 × 10^−17^	intron	*ALDH1A2*	1
HipOA	TJR,THR, KneeHipOA, AllOA	rs12908498	15:67366488	C	G	0.54	1.08	1.06–1.10	1.85 × 10^−16^	intron	*SMAD3*	1
KneeHipOA	TJR, HipOA, KneeOA	rs9940278	16:53800200	T	C	0.43	1.06	1.04–1.07	1.45 × 10^−18^	intron	*FTO*	1
KneeOA	TKR	rs34195470	16:69955690	A	G	0.45	0.95	0.94–0.96	3.13 × 10^−13^	intron	*WWP2*	0
AllOA	TKR, KneeHipOA, KneeOA	rs216175	17:2167690	A	C	0.83	1.04	1.03–1.06	2.74 × 10^−12^	intron	*SMG6*	NA
THR	HipOA	rs7212908	17:59654593	A	G	0.8	0.91	0.89–0.94	1.95 × 10^−11^	intergenic	*NACA2*	0
THR	TJR, HipOA	rs2716212	17:67503653	A	G	0.62	0.93	0.91–0.95	3.56 × 10^−10^	intron	*MAP2K6*	0
AllOA	KneeOA	rs10405617	19:10752968	A	G	0.32	1.03	1.02–1.04	9.33 × 10^−11^	intron	*SLC44A2*	NA
TJR	AllOA	rs75621460	19:41833784	A	G	0.03	1.21	1.14–1.28	2.72 × 10^−10^	intron	*TGFB1*	1
THR	HipOA, TJR	rs4252548	19:55879672	T	C	0.02	1.39	1.29–1.49	2.49 × 10^−19^	Missense p.Arg33His	*IL11*	1
KneeOA	AllOA, TJR, KneeHipOA, TKR	rs143384	20:34025756	A	G	0.59	1.07	1.06–1.09	1.01 × 10^−23^	5_prime_UTR	*GDF5*	1
THR	TJR	rs9981408	21:40017446	T	G	0.23	1.10	1.07–1.12	2.21 × 10^−12^	intron	*ERG*	0

**Female-specific**												

THR		rs116112221	2:59439973	T	C	6.1 × 10^−3^	1.95	1.58–2.41	4.61 × 10^−10^	upstream_gene	*FALCL1*	NA
THR		rs10282983	8:69590554	T	C	0.22	1.15	1.11–1.19	2.21 × 10^−14^	intron	*C3ORF34*	NA
AllOA		rs10453201	9:34050345	T	C	0.22	1.05	1.02–1.06	1.05 × 10^−8^	upstream_gene	*UBAP2*	NA

**Early-onset**												

AllOA		rs148693048	8:24598320	T	C	0.0012	6.26	3.26–12.00	3.37 × 10^−8^	intron	*NEFM*	NA

Abbreviations: osteoarthritis (OA) phenotype, OA phenotype with a genome-wide significant association (p < 1.3 × 10^−8^) with the exception of the early-onset analysis (p < 5 × 10^−8^); other osteoarthritis phenotypes, other OA phenotypes associated with this SNV at GWS level but less significant than OA phenotype; AllOA, OA at any joint site; KneeOA, OA of the knee; HipOA, OA of the hip; KneeHipOA, KneeOA and/or HipOA; TJR, total knee and/or hip replacement; TKR, total knee replacement; THR, total hip replacement; SpineOA, OA of the spine; FingerOA, OA of the finger; ThumbOA, OA of the thumb; HandOA, FingerOA and/or ThumbOA; EA, effect allele; NEA, Non-Effect allele; EAF, effect allele frequency; OR, odds ratio; 95% CI, 95% confidence interval of the OR; Annotation, most severe consequence according to grch37 Ensembl REST API (Yates et al., 2015), if missense the amino acid change is provided according to the Human Gene Mutation Database nomenclature; NearestGene, nearest gene according to grch37 Ensembl REST API; WtGrp, which weight bearing group the signal belongs to (p < 5 × 10^−4^), 0 = weight bearing only, 1 = weight bearing and non-weight bearing, 2 = non-weight bearing only, NA = unclassified due to incomplete information for all phenotypes, a female specific association or an AllOA association.

See also Table S3.

Here, we report signals for spine (n = 1) and thumb (n = 2) osteoarthritis and increase the number of risk SNVs for hand (5 new, 3 previously reported) and finger (3 new, 2 previously reported) osteoarthritis, phenotypes that had not been studied at scale before (Tables 1, 2, and S3). Of the 100 SNVs, 90 are common (minor allele frequency [MAF] ≥5%) and 4 are low-frequency variants (MAF <5% and ≥0.5%). We detected 6 rare variant associations (MAF 0.03%–0.11%) with large effect sizes (odds ratio [OR] range = 3.03–9.52) (Table 2); 1 variant association was previously reported and 5 variant associations are new findings. All of the new rare variant associations are primarily driven by a large extended family in Iceland.

Signals from 4 osteoarthritis phenotypes (spine, knee, knee and/or hip, and osteoarthritis at any site) included individuals of non-European ancestry (between 0.9%–2.8% of cases were of East Asian descent). Even though sample sizes in the East Asian cohorts are small, we observed that 62% of the signals have supportive evidence in East Asian ancestry-only analysis, with the same direction of effect, and 20% of these signals are also nominally significant (binomial test p = 2.27 × 10^−5^, 95% confidence interval [CI] = 7%–100%) (STAR Methods).

We investigated the predictive power of polygenic risk scores (PRS) and found significantly higher odds of developing disease in individuals at the higher decile of the PRS distribution for several osteoarthritis phenotypes (Table S4; STAR Methods).

#### Female-specific osteoarthritis risk variants

To investigate the presence of osteoarthritis signals specific to males only, females only, or with effects of opposite direction in men and women, we performed a sex-differentiated test of association and a test of heterogeneity in allelic effects (Mägi et al., 2010; Mägi and Morris, 2010). We identified 3 new female-specific independent SNVs, two of which showed significant (Phet-diff <0.016) differences in effect size between sexes (Tables 2 and S5). rs116112221 (Psex-diff = 3.20 × 10^−9^, Phet-diff = 4.09 × 10^−4^; female OR = 1.95, 95% CI = 1.58–2.41, P-female = 4.61 × 10^−10^; male OR = 1.06, 95% CI = 0.82–1.38, P-male = 0.64) is significant in the female-only total hip replacement phenotype and is located in a region containing long intergenic non coding RNAs with the closest protein coding gene being *FANCL*. *FANCL* mutations are potentially causative for premature ovary insufficiency in humans (Yang et al., 2020), a condition that leads to early menopause, which has been suggested to be linked to increased prevalence of osteoarthritis, although definitive evidence for this hypothesis is still lacking (Jung et al., 2018; Srikanth et al., 2005). Preclinical and clinical studies indicate that selective estrogen receptor modulators (SERMs) treatment has consistently positive effects on osteoarthritis, especially for postmenopausal patients with early-stage or osteoporotic osteoarthritis (Xiao et al., 2016).

We further identified a signal associated with total hip replacement with opposite direction of effects between men and women, rs10282983 (Psex-diff = 4.93 × 10^−16^, Phet-diff = 7.66 × 10^−14^; female OR = 1.15, 95% CI = 1.11–1.19, P-female = 2.21 × 10^−14^; male OR = 0.92, 95% CI = 0.88–0.96, P-male = 5.16 × 10^−4^). rs10282983 resides in an intron of *C8orf34*, which has been associated with waist-to-hip ratio (Kichaev et al., 2019; Pulit et al., 2019) and heel bone mineral density (Kichaev et al., 2019), both risk factors for osteoarthritis (Hardcastle et al., 2015; Lohmander et al., 2009). rs10453201 is significantly associated with female osteoarthritis at any site (Psex-diff = 5.67 × 10^−9^, Phet-diff = 0.049; female OR = 1.05, 95% CI = 1.03–1.06, P-female = 1.05 × 10^−8^; male OR = 1.02, 95% CI = 1.003–1.04, P-male = 0.02) and is located 5′ of *UBAP2*, which has been associated with Parkinson’s disease (Nalls et al., 2019), type 2 diabetes (Xue et al., 2018), BMI (Kichaev et al., 2019), and heel bone mineral density (Morris et al., 2019) in humans.

#### Early-onset osteoarthritis

Genome-wide meta-analysis identified a new risk variant for early osteoarthritis with large effect size and low allele frequency (rs148693048; effect allele frequency = 0.12%, p = 3.37 × 10^−8^, OR = 6.26, 95% CI = 3.26–12.00) (Tables 2 and S3). The variant is nominally significantly associated in all contributing studies and with the same direction of effect. rs148693048 has not been associated with osteoarthritis before. Two protein-coding genes in the vicinity show significantly different expression levels in intact compared to degraded cartilage (*NEFM* and *DOCK5*). *NEFM* (neurofilament medium) is relevant to the elongation of neuronal structures (Pezzini et al., 2017), and the expressed protein is commonly used as a biomarker of neuronal damage (Khalil et al., 2018). The guanine nucleotide exchange activity of *DOCK5* (dedicator of cytokinesis 5) has been identified as a regulator of osteoclast function, playing an essential role in bone resorption (Vives et al., 2011). Pharmacological inhibition of its activity prevents osteolysis, while preserving bone formation in both humans and mice (Mounier et al., 2020). Intronic variation in *DOCK5* also shows association (p < 5.0 × 10^−8^) with other bone phenotypes, such as heel bone mineral density (Kim, 2018) and adolescent idiopathic scoliosis (Liu et al., 2018).

### Cross-phenotype analysis

#### Similarities and differences of signals across phenotypes

**Figure 2:**
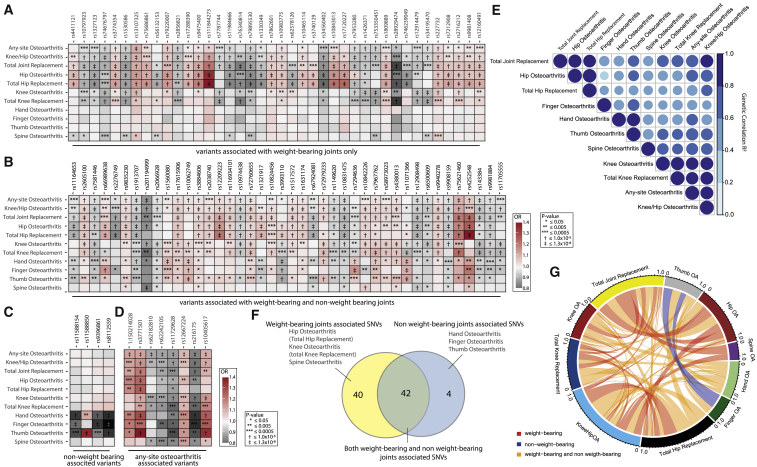
Similarities and differences of signals across phenotypes Correlation and overlap between osteoarthritis genetics (A–D) Heatmap plots of osteoarthritis associated single nucleotide variants (SNVs). Effect sizes (OR, odds ratio) and p values are displayed for each lead SNV for each osteoarthritis phenotype GWAS results. OR are plotted as color, and p values are represented as symbols in the box. (A) Weight bearing joints only (hip, knee, and spine). (B) Both weight and non-weight bearing joints (hip, knee, spine, hand, finger, and thumb). (C) Non-weight bearing joints (hand, finger, and thumb). (D) Any-site osteoarthritis SNVs. (E) Heatmap plot of the genetic correlation (R^2^) between the examined osteoarthritis phenotypes. (F) Venn diagram depicting the number and overlap of SNVs associated with weight bearing and non-weight bearing joints. (G) Circos plot depicting the overlap in osteoarthritis associations of the 100 lead variants. See also Table S6.

We observed that some variants demonstrate a joint-specific effect. We found that the majority of SNVs (60 out of the 100) were genome-wide significantly associated with more than one osteoarthritis phenotype (Figure 2). Forty of the identified SNVs show genome-wide significant associations with weight bearing joints only and 4 SNVs show genome-wide significant associations with non-weight bearing joints only (Figure 2; Table S3). We have over 80% power to detect all 4 non-weight bearing specific variants in the weight bearing joint analyses (at genome-wide significance). Further, we have over 80% power to detect 22 of the 40 weight bearing joint-specific effects in non-weight bearing joint analyses (hand osteoarthritis). Although several core pathways are known to underpin osteoarthritis pathology, regardless of joint site affected, no common genetic osteoarthritis SNVs have been found previously, with the exception of the *GDF5* locus (Reynard and Loughlin, 2013; Sandell, 2012). Here, we have identified 42 SNVs with strong association across both weight bearing and non-weight bearing joints. Several of these SNVs, rs3771501 (*TGFA*), rs3993110 (*TEAD1/DKK3*), rs72979233 (*CHRDL*2), and rs7967762 (*PFKM*/*WNT10B*) (Figures 2B and 2D), are associated with multiple osteoarthritis joint sites. These signals likely represent a common underlying mechanism in osteoarthritis pathology. They have been shown to play a role in the transforming growth factor β (TGF-β)/bone morphogenetic protein (BMP), Wnt/β-catenin signaling pathways, the functional interaction of which has been implicated in the pathogenesis of osteoarthritis (Wu et al., 2012). These signaling pathways could be prime candidates for drug development.

Additional insights may also be gleaned from the comparison of association signals across osteoarthritis phenotypes. Most of the SNVs associated with knee, hip, and knee and/or hip osteoarthritis have a larger effect size on the respective joint replacement-defined phenotypes, all of which are notably of smaller sample size. This could be driven by homogeneity of phenotype definition (Manchia et al., 2013) (Table S1) or can represent a biological and functional relevance, indicating that these loci might play more important roles in receiving a joint replacement (i.e., pain and inflammation) than in osteoarthritis pathology itself. For example, rs76340814 (*PTCH1*) and rs28929474 (missense variant in *SERPINA1*) have stronger associations and larger effect sizes with total hip replacement (THR), total knee replacement (TKR), and total joint replacement (TJR), than with hip or knee osteoarthritis (Figure 2A). Indeed, *PTCH1* is thought to function in neurogenic and brain development (Mansilla et al., 2006; Ribeiro et al., 2010), and *SERPINA1* is thought to function in inflammation. Studies in rat osteoarthritis models have shown that early treatment with alpha-1-antiproteinase, encoded by *serpina1*, blocked the proteolytic activity of neutrophil elastase and caused lasting improvements in joint inflammation, pain, and saphenous nerve damage (Muley et al., 2017).

#### Genetic links between phenotypes

We found osteoarthritis subtypes to share substantial genetic components, albeit with a wide range (Figure 2E; Table S6).

We investigated if osteoarthritis genetic components are shared with other traits and found significant correlation with anthropometric traits (BMI, obesity, weight, and fat mass), type 2 diabetes, education, depressive symptoms, smoking behavior, bone mineral density, reproductive phenotypes and intelligence as previously reported (Tachmazidou et al., 2019; Zengini et al., 2018), and several pain phenotypes (Table S6).

Pain is the most disabling symptom experienced by osteoarthritis patients and is one of the main reasons to proceed to physician consultation and total joint replacement (Schaible, 2018). The etiology of pain in osteoarthritis is multifactorial including significant soft tissue inflammation, the sensitization of pain pathways involving the joint nociceptors, the nociceptive processing in the CNS, and neuropathic pain components in osteoarthritis models (Dimitroulas et al., 2014; Fu et al., 2018; Hsia et al., 2018; Kidd, 2012). Although a main symptom, no genetic determinants of osteoarthritis pain have been discovered before. We found high correlation between osteoarthritis and sciatica, fibromyalgia, headaches, and other back pain phenotypes, where the highest correlation is with spine osteoarthritis (genetic correlation [rg] = 0.61, 0.87, 0.39, and 0.79, respectively). *SOX5*, one of the new signals, has been previously reported to be upregulated in human osteoarthritis cartilage (Liu et al., 2020) and has been associated with back pain and with lumbar intervertebral disc degeneration (Suri et al., 2018). These findings are supported by animal model data, in which inactivation of SOX5 leads to defects in skeletogenesis such as in cartilage development, the notochord, and intervertebral discs in mice (Smits and Lefebvre, 2003; Smits et al., 2001). We also observed strong correlation between osteoarthritis and pain phenotypes in the LD-Hub database (all derived from the UK Biobank dataset), in particular between spine osteoarthritis and dorsalgia (rg = 0.87), leg pain on walking (rg = 0.82), knee pain (rg = 0.63), hip pain (rg = 0.76), back pain (rg = 0.75), and neck/shoulder pain (rg = 0.67) (Table S6). Thus, our data suggest that a proportion of the identified signals are also associated with osteoarthritis pain.

### Effector genes and biological pathways

#### Identification of putative causal variants

**Figure S1:**
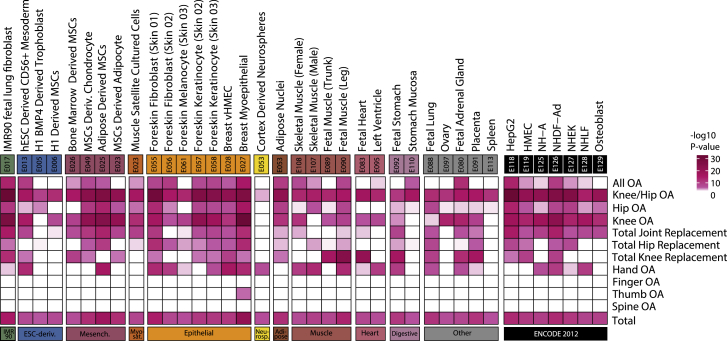
Identification of involved tissues, related to Effector genes and biological pathways and STAR Methods Heatmap depicting tissue-specific gene-regulatory region enrichment significance (-log10 P value) for all osteoarthritis GWAS phenotypes. Tissue/cell type (full name, E-identifier, group name) and P value (-log10) of all significant enrichments (p < 1.3x10^−8^) are shown. Enrichment was calculated using all osteoarthritis associated lead SNVs and the fine-mapped variants, per osteoarthritis phenotype and all together. Only rows and columns containing a significant enrichment (p < 1.3x10^−8^) for all osteoarthritis phenotypes (Total) are shown. OA: osteoarthritis.

We employed complementary computational approaches (STAR Methods) to fine-map the GWAS signals to a small set of likely causal variants, identify relevant tissues based on signal enrichment (Figure S1), and provide mechanistic insights based on expression quantitative trait locus (eQTL) colocalization and causal inference analysis (Table S7). Twelve signals were fine-mapped to variant sets contained entirely within the transcript of a single gene with >95% posterior probability (PP), although we note that this does not provide conclusive evidence for the effector gene. Of note, *ALDH1A2*, which fine-maps to 6 intronic variants with 99% PP, is currently the target of approved drugs in use for other indications, providing a potential opportunity for drug repositioning (Sumita et al., 2017) (Table S8).

For 6 SNVs (3 new and 3 known), a single variant could be postulated as causal with >95% PP (Table S8).

#### Amassing evidence to identify effector genes

We assessed if any of the genes residing within 1 Mb of the osteoarthritis-associated lead variants showed differential gene expression and protein abundance in primary osteoarthritis-affected tissue in chondrocytes extracted from osteoarthritis patients undergoing joint replacement surgery. Similarly, we compared gene expression of subchondral bone tissue underneath the intact and degraded cartilage tissue (Tables S9 and S10). By combining results from the complementary functional genomics and computational approaches (outlined above), we identified 637 genes with at least one line of evidence pointing to a putative effector gene (Table S10). For these 637 genes, we combined supportive information from the fine-mapping, eQTL colocalization analyses, animal model data, human musculoskeletal and neuronal phenotype data, functional genomics, and causal inference analysis and identified 77 genes that have at least 3 different lines of evidence in support of their role as an effector gene (Tables 3 and S10). Of these 77 genes, 4 are supported by missense lead variants (rs2276749 in *VGLL4*, rs3740129 in *CHST3*, rs143083812 in *SMO*, and rs4252548 in *IL11*). Forty eight provide strong additional evidence for the likely effector gene at previously reported osteoarthritis-associated SNVs (Table 3) and 30 reside in newly associated signals.

**Table 3:** Amassing evidence to identify effector genes

Signal	Lead OA SNV	EA	EAF	OA	Coding variant and fine map	Fine mapped gene	eQTL colocalization (Gtex/OA tissue)	Cartilage		Bone	Blood pQTL MR+coloc	Mouse musculoskeletal phenotype	Human musculoskeletal phenotype	Mouse neuronal phenotype	Human pain disorder	Human pain gene	Score
								Expr.	Abund.	Expr.							
9	rs3740129	A	0.46	N	*CHST3*		*CHST3* (6/1)					*CHST3*	*CHST3*	*CHST3*			6
33	rs12908498	C	0.54	K		*SMAD3*	*SMAD3* (1/1)					*SMAD3*	*SMAD3*			*SMAD3*	6
54	rs143384	A	0.59	K		*GDF5*	*GDF5* (4/1)					*GDF5*	*GDF5*			*GDF5*	6
14	rs67924081	A	0.74	K			*LTBP3* (1/1)		LTBP3 (+)			*LTBP3*	*LTBP3*				5
22	rs7294636	A	0.37	K			*MGP* (4/2)				MGP	*MGP*	*MGP*				5
25	rs7967762	T	0.16	N				*WNT10B* (+)		*WNT10B* (+)		*WNT10B*	*WNT10B*	*WNT10B*			5
49	rs66989638	A	0.13	N	*C2orf40*	*C2orf40*	*C2orf40* (40/21)							*C2orf40*			5
69	rs1530586	T	0.8	K			*FGFR3* (6/4)					*FGFR3*	*FGFR3*	*FGFR3*			5
72	rs17615906	T	0.84	N			*FBN2* (2/2)					*FBN2*	*FBN2*	*FBN2*			5
97	rs62578126	T	0.37	K			*LMX1B* (3/2)					*LMX1B*	*LMX1B*	*LMX1B*			5
17	rs1149620	A	0.44	K		*TSKU*	*TSKU* (5/4)							*TSKU*			4
25	rs7967762	T	0.16	N			*COL2A1* (1/0)					*COL2A1*	*COL2A1*	*COL2A1*			4
25	rs7967762	T	0.16	N			*PFKM* (2/2)		PFKM (–)			*PFKM*					4
25	rs7967762	T	0.16	N								*VDR*	*VDR*	*VDR*		*VDR*	4
28	rs58973023	A	0.49	N				*TNFSF11* (+)		*TNFSF11* (+)		*TNFSF11*	*TNFSF11*				4
31	rs11071366	A	0.61	K		*ALDH1A2*			ALDH1A2 (–)			*ALDH1A2*		*ALDH1A2*			4
33	rs12908498	C	0.54	K								*MAP2K1*	*MAP2K1*	*MAP2K1*		*MAP2K1*	4
34	rs12914479	C	0.66	N			*IGF1R* (1/1)					*IGF1R*		*IGF1R*			4
35	rs6500609	C	0.11	N			*HMOX2* (1/1)					*HMOX2*		*HMOX2*			4
42	rs2716212	A	0.62	K					PRKAR1A (–)			*PRKAR1A*	*PRKAR1A*	*PRKAR1A*			4
45	rs75621460	A	0.03	K		*TGFB1*						*TGFB1*	*TGFB1*			*TGFB1*	4
47	rs4252548	T	0.02	K	*IL11*	*IL11*		*IL11 (+)*		*IL11* (+)							4
53	rs3771501	A	0.47	K		*TGFA*	*TGFA* (2/2)					*TGFA*					4
55	rs9981408	T	0.23	K		*ERG*	*ERG* (1/1)					*ERG*					4
59	rs2276749	T	0.05	N	*VGLL4*	*VGLL4*	*VGLL4* (1/0)					*VGLL4*					4
93	rs1330349	C	0.59	K		*TNC*		*TNC* (+)	TNC (+)					*TNC*			4
100	rs76340814	A	0.05	N			*PTCH1* (5/2)					*PTCH1*		*PTCH1*			4
1	rs11164653	T	0.41	K		*COL11A1*						*COL11A1*	*COL11A1*				3
3	1:150214028	D	0.38	K								*CTSK*	*CTSK*	*CTSK*			3
3	1:150214028	D	0.38	K								*SF3B4*	*SF3B4*	*SF3B4*			3
5	rs1327123	C	0.35	N	*TSEN15*		*TSEN15* (8/5)										3
6	rs2605100	A	0.32	K								*IARS2*	*IARS2*		*IARS2*		3
7	rs11588850	A	0.82	N			*SNAP47* (3/3)					*SNAP47*					3
11	rs3993110	A	0.61	N		*TEAD1*						*TEAD1*		*TEAD1*			3
18	rs10831475	A	0.81	N				*MTMR2 (+)*				*MTMR2*		*MTMR2*			3
24	rs10843013	A	0.78	K								*PTHLH*	*PTHLH*	*PTHLH*			3
25	rs7967762	T	0.16	N								*WNT1*	*WNT1*	*WNT1*			3
30	rs4380013	A	0.19	K								*CYP19A1*		*CYP19A1*		*CYP19A1*	3
30	rs4380013	A	0.19	K		*USP8*	*USP8* (6/2)										3
35	rs6500609	C	0.11	N								*CREBBP*	*CREBBP*	*CREBBP*			3
36	rs9940278	T	0.43	K		*FTO*						*FTO*		*FTO*			3
36	rs9940278	T	0.43	K								*RPGRIP1L*	*RPGRIP1L*	*RPGRIP1L*			3
37	rs34195470	A	0.45	K		*WWP2*		*WWP2* (–)				*WWP2*					3
38	rs216175	A	0.83	K								*BHLHA9*	*BHLHA9*	*BHLHA9*			3
38	rs216175	A	0.83	K								*SERPINF1*	*SERPINF1*	*SERPINF1*			3
39	rs227732	T	0.3	N								*NOG*	*NOG*	*NOG*			3
40	rs9908159	T	0.51	N								*NOG*	*NOG*	*NOG*			3
41	rs7212908	A	0.8	K				*TBX4* (–)				*TBX4*	*TBX4*				3
42	rs2716212	A	0.62	K		*MAP2K6*	*MAP2K6* (1/1)										3
44	rs10405617	A	0.32	K			*ILF3* (2/1)					*ILF3*					3
44	rs10405617	A	0.32	K								*SMARCA4*	*SMARCA4*	*SMARCA4*			3
45	rs75621460	A	0.03	K								*ERF*	*ERF*	*ERF*			3
45	rs75621460	A	0.03	K								*MEGF8*	*MEGF8*	*MEGF8*			3
45	rs75621460	A	0.03	K								*SPTBN4*		*SPTBN4*	*SPTBN4*		3
46	rs8112559	C	0.89	N								*APOE*		*APOE*		*APOE*	3
51	rs7581446	T	0.48	K		*LTBP1*	*LTBP1 (1/0)*					*LTBP1*					3
55	rs9981408	T	0.23	K								*KCNJ6*		*KCNJ6*		*KCNJ6*	3
58	rs12160491	A	0.71	N			*TRIOBP* (22/13)	*TRIOBP* (–)									3
69	rs1530586	T	0.8	K								*IDUA*	*IDUA*	*IDUA*			3
69	rs1530586	T	0.8	K			*TACC3* (9/6)					*TACC3*					3
73	rs10062749	T	0.27	N								*NR3C1*		*NR3C1*		*NR3C1*	3
74	rs3884606	A	0.52	K		*FGF18*		*FGF18* (+)				*FGF18*					3
74	rs3884606	A	0.52	K								*SH3PXD2B*	*SH3PXD2B*	*SH3PXD2B*			3
76	rs56132153	A	0.61	N				*PIK3R1* (–)				*PIK3R1*		*PIK3R1*			3
77	rs9396861	A	0.61	N		*RNF144B*	*RNF144B* (2/1)										3
78	rs79220007	T	0.93	K	*HFE*							*HFE*		*HFE*			3
81	rs17288390	T	0.65	K								*CLIC5*		*CLIC5*		*CLIC5*	3
81	rs17288390	T	0.65	K								*RUNX2*	*RUNX2*			*RUNX2*	3
82	rs9475400	T	0.1	K								*HCRTR2*		*HCRTR2*		*HCRTR2*	3
83	rs12209223	A	0.11	K					MYO6 (+)			*MYO6*		*MYO6*			3
84	rs116934101	A	0.27	N		*CUX1*		*CUX1* (+)				*CUX1*					3
86	rs143083812	T	0.0011	K	*SMO*							*SMO*		*SMO*			3
87	rs571734653	A	3.00E-04	N								*CHRM2*		*CHRM2*		*CHRM2*	3
88	rs7787744	A	0.67	N								*NOS3*		*NOS3*		*NOS3*	3
89	rs111844273	A	0.02	K								*TWIST1*	*TWIST1*	*TWIST1*			3
92	rs72760655	A	0.33	K				*COL27A1* (–)				*COL27A1*	*COL27A1*				3
93	rs1330349	C	0.59	K				*COL27A1* (–)				*COL27A1*	*COL27A1*				3
96	rs10983775	T	0.54	N								*TLR4*		*TLR4*		*TLR4*	3
98	rs10465114	A	0.22	N								*LMX1B*	*LMX1B*	*LMX1B*			3
S1	rs10453201	T	0.22	N				*ENHO* (–)				*ENHO*		*ENHO*			3

Abbreviations: Lead OA SNV, rsID of the lead variant; EA, effect allele; EAF, effect allele frequency; OA, if the signal is new (N) or previously reported (K); Coding variant and FineMap, gene in which the lead SNV or a SNV in high LD (R^2^ ≥0.8) has a moderate to high severity consequence (STAR Methods) and is present in the 95% credible set (CS); Fine-mapped gene, all SNVs in the 95% CS reside within the transcript of the gene; eQTL colocalization, gene colocalized in at least 1 GTEx tissue, the number of GTEx tissues in parentheses followed by the number of these tissues also enriched in tissue enrichment analysis, which is suggestive of a role in osteoarthritis pathology; Cartilage Differential Expr, gene was differentially expressed (+ indicates increased, – indicates decreased) in high-grade compared to low-grade osteoarthritic cartilage; Cartilage Differential Abund, gene that codes for a protein that was differentially expressed (+ indicates increased, – indicates decreased) in high-grade compared to low-grade osteoarthritic cartilage; Bone Differential Expr, gene was differentially expressed in subchondral bone; Blood pQTL MR+coloc, gene is on the causal path and also colocalized; Human musculoskeletal phenotype, gene linked to a musculoskeletal phenotype according to the nosology and classification of genetic skeletal disorders (Mortier et al., 2019); Mouse musculoskeletal/Neuronal phenotype, indicates if a musculoskeletal (skeleton, limb/digit/tail, and muscle) or neuronal phenotype (“nervous system phenotype” included in the MGI mouse phenotype ontology) is observed in any mouse knockout from https://www.hugedomains.com/domain_profile.cfm?d=boneandcartilage&e=com and the MGI Mouse Genome Informatics from http://www.informatics.jax.org/; Human pain disorder, gene is linked to a pain or neuropathy disorder according to OMIM (https://www.omim.org/); Human Pain Gene, gene is linked to pain in the Human Pain Genetics Database (HPGD) (http://humanpaingenetics.org/hpgdb; Score, cumulative score for each gene based on the supporting fine-mapping and functional analysis. Genes are identified by Ensembl GeneName. See also Tables S9 and S10.

*CHST3*, *SMAD3*, and *GDF5* accrued the highest levels of confidence, each with 6 different lines of evidence in support of their involvement in osteoarthritis. *CHST3* (carbohydrate sulfotransferase 3) represents a newly identified signal and encodes chondroitin sulfate, the major proteoglycan present in cartilage. Mutations in *CHST3* have been previously associated with short stature, congenital joint dislocations, clubfoot, Larsen syndrome, and elbow joint dysplasia (Superti-Furga and Unger, 1993; Unger et al., 2010). *CHST3* has also been shown to be associated with lumbar disc degeneration (Song et al., 2013).

**Figure 3:**
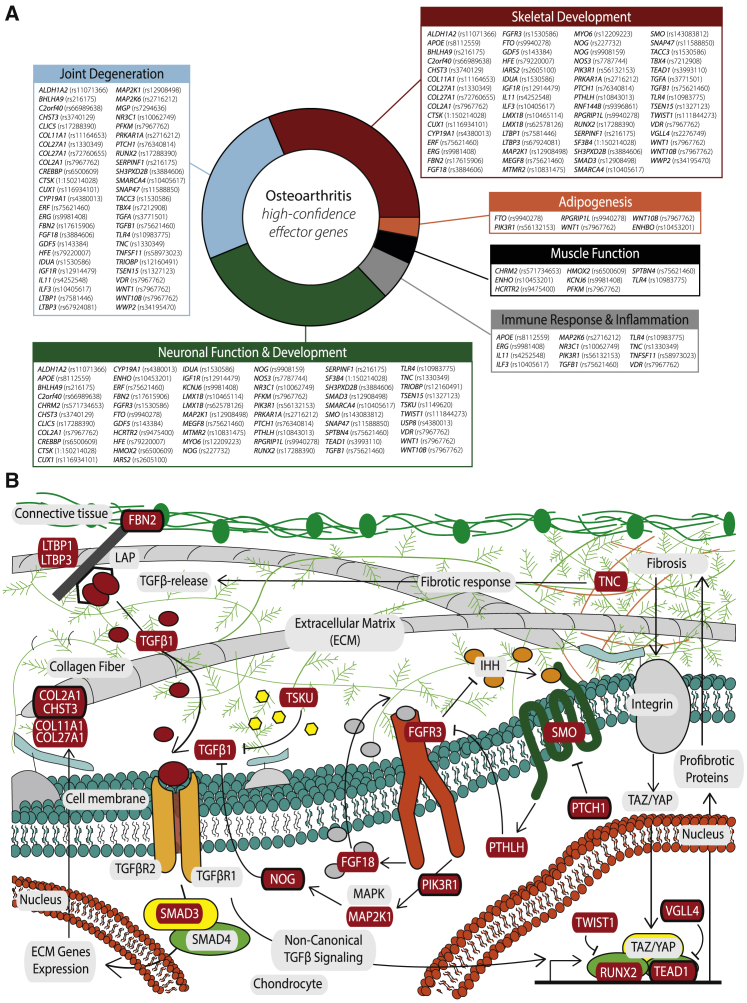
High-confidence osteoarthritis effector genes (A) Overview of the 77 high-confidence osteoarthritis effector genes and their broad biological classifications, as depicted in Tables 3 and S12. The lead SNV for each is given in brackets. (B) Schematic representation of a chondrocyte and its extracellular matrix, highlighting exemplary osteoarthritis-implicated biological pathways (TGF-β signaling, FGFR3 signaling, and part of the fibrosis pathway) and the high-confidence effector genes (in red boxes), both established and newly identified (in red boxes with a black outline) that have been found to play a role.

To glean further insight into the biological role of the high-confidence effector genes in disease processes, we integrated additional information based on the analysis of endophenotypes more closely related to the underlying biology, monogenic and rare human disease data, phenome-wide analyses, and additional functional genomics data (Tables S11 and S12; STAR Methods). By synthesizing all lines of evidence, we found that the assignment of several of the 77 high-confidence effector genes into likely mechanisms through which they exert their effect traverses multiple biological processes (Figure 3A). Here, we primarily focus on the newly associated genes that are reported in this work. These represent high-value candidates for further mechanistic and clinical investigation.

The majority of high-confidence effector genes are associated with skeletal development (63 in total, 21 genes associated with newly reported signals) and joint degradation (50 in total, 18 genes associated with newly reported signals; 13 genes in common between the skeletal development and joint degradation categories) (Figure 3A). Three effector genes arising from new genetic signals encode structural proteins: CHST3, COL2A1, and FBN2. Collagen type II alpha 1 chain (*COL2A1*) codes for an essential structural component of cartilage and is important for joint formation and bone growth (Figure 3B). A wide spectrum of diseases is associated with *COL2A1*, including cartilage and bone abnormalities, such as spondyloepimetaphyseal dysplasia, Kniest dysplasia, and early onset osteoarthritis (Kuivaniemi et al., 1991; Löppönen et al., 2004; Wilkin et al., 1999; Xiong et al., 2018). Fibrillin 2 (*FBN2*) encodes a glycoprotein that forms microfibrils in the extracellular matrix and has a major role during early morphogenesis. Fibrillins potently regulate pathways of the immune response, inflammation, and tissue homeostasis (Zeyer and Reinhardt, 2015), are important in bone remodeling, and regulate local availability of BMP and TGF-β (Nistala et al., 2010) (Figure 3B). Mutations in *FBN2* cause contractual arachnodactyly (Putnam et al., 1995).

Several genes are connected with signaling pathways. Vestigial like family member 4 (*VGLL4*) functions via interacting with TEA domain (TEAD) transcription factors (Jiao et al., 2017; Lin et al., 2016). Notably, we identified another new THR and hand osteoarthritis-associated signal located in such a transcription factor, the *TEAD1* gene, indicating a common molecular pathway underlying both signals (Figure 3B). TEAD1 functions in the Hippo signaling pathway and is transcriptionally regulated by the YAP1 and TAZ protooncogene proteins, which are involved in mechanosensing and mechanotransduction (Dupont et al., 2011; Low et al., 2014). Mechanoadaptation of articular cartilage is an important factor in osteoarthritis (Vincent and Wann, 2019; Zhao et al., 2020). Downregulation of *VGLL4* is linked to the upregulation of Wnt/β-catenin pathway target genes (Jiao et al., 2017).

Wnt family member 1 (*WNT1*) and wnt family member 10B (*WNT10B*) are involved in the Wnt signaling pathway, which has an established role in osteoarthritis pathogenesis (Zhou et al., 2017). Mutations in *WNT10B* have been linked to limb defects and dental abnormalities (Kantaputra et al., 2018; Ullah et al., 2018; Yu et al., 2016), and mutations in *WNT1* are associated with osteogenesis imperfecta (Fahiminiya et al., 2013). Insulin like growth factor 1 receptor (*IGF1R*) has tyrosine kinase activity, mediates the action of insulin-like growth factor, and regulates cartilage mineralization (Heilig et al., 2016).

Nitric oxide synthase 3 (*NOS3*) encodes the vascular endothelium isoform of nitric oxide synthase (eNOS). *NOS3* is associated with sporadic limb defects in mice (Gregg et al., 1998) and has been implicated in bone remodeling in rats (Hukkanen et al., 1999). LIM homeobox transcription factor 1 beta (*LMX1B*) is a transcription factor. Mutations in *LMX1B* cause a rare autosomal dominant disorder characterized by dystrophic nails, hypoplastic or absent patellae, and dysplasia of the elbows and iliac horn (Marini et al., 2010).

Patched 1 (*PTCH1*) codes for a receptor for Hh ligands and regulates the activity of smoothened, frizzled class receptor (*SMO*,another effector gene associated with a known lead SNV). When bound, PTCH1 relinquishes its inhibitory effect on SMO and activates the Hh signaling cascade, which plays an important role in controlling the proliferation of chondrocytes and also in stimulating osteogenesis during endochondral bone formation and longitudinal growth (Alman, 2015).

Several further newly identified high-confidence effector genes have a neuronal connection (Figure 3A). Augurin, the protein encoded by *C2orf40* (also called *ECRG4*), is involved in CNS development in animal models (Gonzalez et al., 2011) and shows association with neuropathologic features of Alzheimer’s disease and related dementias in humans (Beecham et al., 2014). SNVs in the vicinity of *TSEN15* have been robustly associated with anthropometric traits that have epidemiological links to osteoarthritis, such as height (Gudbjartsson et al., 2008), body fat distribution (Rask-Andersen et al., 2019), and waist circumference adjusted for BMI (Hübel et al., 2019). Cut like homeobox 1 (*CUX1*) is a transcription factor involved in brain neuronal differentiation and synaptogenesis (Cubelos et al., 2010). *Cux1* expression was observed at chondrogenic interzones during limb development, suggesting also a regulatory role in joint formation (Lizarraga et al., 2002).

The TRIO and f-actin binding protein (*TRIOBP*) gene encodes multiple protein isoforms via 2 promoters (Park et al., 2018). *TRIOBP*-*1* is ubiquitously expressed and interacts with TRIO and f-actin binding protein that together play crucial roles in neuronal morphogenesis (Woo et al., 2019) and controlling actin cytoskeleton organization, cell motility, and cell growth (Zaharija et al., 2020).

Myotubularin related protein 2 (*MTMR2*) has an important role in membrane targeting, vesicular trafficking, and regulation of signal transduction pathways. Mutations in *MTMR2* cause Charcot-Marie-Tooth disease type 4B, which features a generalized loss of large myelinated nerve fibers and focally folded myelin sheaths giving rise to inadequate nerve signaling to muscles, resulting in muscle weakness and atrophy (Volpatti et al., 2019). The ubiquitously expressed protein encoded by CREB-binding protein (*CREBBP*) plays a critical role during development in particular with brain size regulation, correct neural cell differentiation, and neural precursor cell migration, as demonstrated in mouse models (Schoof et al., 2019).

Cholinergic receptor muscarinic 2 (*CHRM2*) is involved in the mediation of cellular responses. Analysis of rat tissues revealed expression in whole brain (Peralta et al., 1987) and in human neuroblastoma cells (Zhou et al., 2001). Variation in *CHRM2* predisposes to various neuropsychiatric diseases (Cannon et al., 2011; Rajji et al., 2012), and Alzheimer’s disease (Mash et al., 1985). The protein encoded by synaptosome associated protein 47 (*SNAP47*) is a soluble N-ethylmaleimide-sensitive fusion protein attachment protein receptor (SNARE) protein involved in trafficking and membrane fusion. SNARE-mediated fusion is an essential mechanism that drives the synaptic transmission, neuron development, and growth. SNAP47 plays a role in exocytic mode and neuronal morphogenesis (Holt et al., 2006; Urbina et al., 2021).

Several of the effector genes have an immune or inflammatory role. For example, the protein encoded by toll like receptor 4 (*TLR4*) plays a fundamental role in pathogen recognition and activation of the innate immune response (Tatematsu et al., 2016). TLR4 is also activated by host-derived molecules generated by damaged tissues related to different musculoskeletal pathologies (Abdollahi-Roodsaz et al., 2007; Goldring and Goldring, 2007). This, along with gene expression in chondrocytes (Wang et al., 2011), osteoblasts (Kikuchi et al., 2001), and synoviocytes (Midwood et al., 2009), has linked *TLR4* to diseases like rheumatoid arthritis (Abdollahi-Roodsaz et al., 2007), osteoarthritis (Gómez et al., 2015), and osteoporosis (Vijayan et al., 2014), where modulation or inhibition of *TLR4* has been suggested as a treatment. Activation of T cells can lead to osteoclastogenesis and bone resorption by influencing the expression of tumor necrosis factor ligand superfamily member 11 (*TNFSF11*) (Kong et al., 1999). *TNFSF11* encodes receptor activator of nuclear factor kappa-β ligand (also known as RANKL), a cytokine that has been linked to inflammatory bone remodeling in rheumatoid arthritis, with increased TNFSF11 levels associated with worsening arthritis severity (Papadaki et al., 2019; Remuzgo-Martínez et al., 2016) and a well-established role in osteoclastogenesis (Kohli and Kohli, 2011).

Nuclear receptor subfamily 3 group C member 1 (*NR3C1*) encodes the glucocorticoid receptor (GR) which circulates in the cytoplasm and is involved in the inflammatory response (Escoter-Torres et al., 2019). In osteoarthritis, endogenous glucocorticoid signaling in osteoblasts and chondrocytes is detrimental (Macfarlane et al., 2020).

Phosphofructokinase (*PFKM*) has a role in muscle function. It encodes a muscle isozyme that catalyzes the phosphorylation of fructose-6-phosphate during glycolysis. Mutations in this gene result in Tarui’s disease (glycogen storage disease type 7) that is an autosomal recessive metabolic disorder characterized clinically by exercise intolerance, muscle cramping, exertional myopathy, and compensated hemolysis (Raben and Sherman, 1995).

### Drug target identification

We examined the druggability status of all 637 genes with at least one piece of supporting evidence from fine-mapping and functional analyses (Table S10; STAR Methods). Of these 637 genes, 205 were present in the druggable genome database (Finan et al., 2017), showing a 1.46-fold enrichment of genes with supporting evidence in the database (binomial test p = 2.21 × 10^−9^) (STAR Methods). From these osteoarthritis druggable target genes, 71 genes reside in tier 1, which incorporates the targets of approved (licensed) drugs and drugs in clinical development (Table S10; STAR Methods). Of the 77 genes with three different lines of evidence supporting causality, 20 are tier 1 candidates (18 of these are present in DrugBank) (Table 4; STAR Methods), of which 7 correspond to new genetic signals discovered in this study (*CHST3*, *VDR*, *TNFSF11*, *IGF1R*, *NR3C1*, *CHRM2*, and *NOS3*).

**Table 4:** Drug repurposing opportunities

Gene	Encoded protein	Uniprot ID	Drug name	Drugbank ID	Molecule type	Development phase	Molecular mechanism of action	Mechanism of action	Current clinical indication(s)
*AAK1*	AP2-associated protein kinase 1	Q2M2I8	Fostamatinib	DB12010	small molecule	approved, investigational	antagonist	inhibitor of spleen tyrosine kinase	chronic immune thrombocytopenia
*ABCB8*^b^	mitochondrial potassium channel ATP-binding subunit	Q9NUT2	Doxorubicin	DB00997	small molecule	approved, investigational	antagonist	cytotoxic anthracycline antibiotic, binds to nucleic acids and inhibits topoisomerase II to exert antimitotic activity	wide range of hematological and non-hematological malignancies
*ADAM10*	disintegrin and metalloproteinase domain-containing protein 10	O14672	XL784	DB04991	small molecule	investigational	antagonist	potent small molecule inhibitor of the ADAM-10 metalloprotease enzyme, which plays a role in blood vessel formation and cell proliferation that can cause renal fibrosis and impairment	investigational in albuminaemia/diabetic nephropathy
*ADRBK1*	beta-adrenergic receptor kinase 1	P25098	ATP	DB00171	small molecule	investigational, nutraceutical	agonist	specifically phosphorylates the agonist-occupied form of the beta-adrenergic and closely related receptors	nutritional supplement, investigational in advanced cancer and in venous stasis ulcers
*ALDH1A2*^c^	retinal dehydrogenase 2	O94788	Tretinoin^a^	DB00755	small molecule	approved	agonist	cell reproduction, proliferation, differentiation	acne, skin keratinization disorders
*APH1A*	gamma-secretase subunit APH-1A	Q96BI3	E-2012	DB5171	small molecule	investigational	antagonist	inhibits beta-amyloid production through inhibition of gamma secretase activity	investigational in Alzheimer’s disease
*ATP1A3*	Sodium/potassium-transporting ATPase subunit alpha-3	P13637	Ouabain	DB01092	small molecule	approved	inhibitor	cardiac glycoside, inhibits the Na-K-ATPase membrane pump	atrial fibrillation, atrial flutter and heart failure
*CACNA1D*	voltage-dependent L-type calcium channel subunit alpha-1D	Q01668	Nimodipine^a^	DB00393	small molecule	approved, investigational	antagonist	voltage-gated calcium channel blocker, inhibiting vascular smooth muscle contraction	Hypertension, including intracranial
*CDK5*^b^	cyclin-dependent-like kinase 5	Q00535	Trilaciclib^a^	DB15442	small molecule	approved, investigational	antagonist	inhibits several CDKs (proline-directed serine/threonine-protein kinases) essential for neuronal cell cycle arrest, most notably CDK4	bone marrow suppression caused by chemotherapy
*CDK7*^b^	cyclin-dependent kinase 7	P50613	Trilaciclib^a^	DB01085	small molecule	approved, investigational	antagonist	inhibitor of serine/threonine kinase involved in cell cycle control and RNA polymerase II-mediated RNA transcription (main action against CDK4 and CDK6, also active against CDK7)	bone marrow suppression caused by chemotherapy
*CHRM2*^b^^,^^c^	muscarinic acetylcholine receptor M2	P08172	Pilocarpine	DB155443	small molecule	approved, investigational	agonist	slowly hydrolyzed muscarinic agonist	dry mouth, ocular hypertension
			Atropine	DB00572	small molecule	approved	antagonist	inhibits the muscarinic action of acetylcholine in postganglionic cholinergic nerves	reduce airway secretions during anesthesia, reduces smooth muscle spasm, increases heart rate, used as antidote to cholinergic acting poisons
*CHST3*^b^^,^^c^	carbohydrate sulfotransferase 3	Q7LGC8	Thalidomide	DB01041	small molecule	approved, withdrawn for hypnotic indications	agonist	modulates cytokine release, catalyzes sulfation of chondroitin	immunosuppressive, anti-angiogenic, experimental in osteoarthritis
*CSNK1E*^b^	casein kinase I isoform epsilon	P49674	Umbralisib^a^	DB14989	small molecule	approved, investigational	antagonist	Umbralisib inhibits several protein kinases, including PI3Kδ and casein kinase CK1ε. PI3Kδ is expressed in both healthy cells and malignant B cells; CK1ε is believed to be involved in the pathogenesis of malignant cells, including lymphomas	relapsed and refractory lymphoma
*CTSK*^c^	cathepsin K	P43235	MIV-711	DB15599	small molecule	investigational	antagonist	osteoclast inhibitor	investigational in osteoarthritis
*CYP19A1*^c^	cytochrome P450 19A1	P11511	Aminoglutethimide^a^	DB00357	small molecule	approved	antagonist	aromatase inhibitor, blocks conversion of androgens to estrogens	breast cancer, prostate cancer
*EGLN2*	Egl nine homolog 2	Q96KS0	Ascorbic acid^a^	DB00126	small molecule	approved	agonist	co-factor in collagen synthesis, carbohydrate, and lipid metabolism; antioxidant.	vitamin C deficiency, investigational in osteoarthritis
*ENG*^b^	endoglin	P17813	Carotuximab	DB06322	small molecule	investigational	unknown	regulator of angiogenesis through TGFB type 2 receptor binding	investigational treatment of solid tumors
*EPHA5*^b^	ephrin type-A receptor 5	P54756	Fostamatinib^a^	DB12010	small molecule	approved, investigational	antagonist	reduces ATP binding to inhibit to ephrin-a family tyrosine kinase	rheumatoid arthritis, immune thrombocytopenic purpura
*EPOR*	erythropoietin receptor	P19235	Erythropoietin^a^	DB00016	recombinant protein	approved	agonist	erythropoietin or exogenous epoetin alfa binds to the erythropoietin receptor (EPO-R) and activates intracellular signal transduction pathways	treatment of anemia
*FGF18*^c^	fibroblast growth factor 18	O76093	Sprifermin	DB12616	recombinant protein	investigational	agonist	cell morphogenesis, chondrogenesis, cartilage thickening in OA	osteoporosis, cancer bone metastasis, investigational in osteoarthritis
*FGFR3*^c^	fibroblast growth factor receptor 3		Pemigatinib^a^	DB15102	small molecule	approved, investigational	antagonist	inhibitor of FGF receptors 1 to 4 that are tyrosine kinases that activate signaling pathways in tumor cells	advanced cholangiocarcinoma in patients with FGFR alterations
*GAK*	cyclin-G-associated kinase	O14976	Fostamatinib	DB12010	small molecule	approved, investigational	antagonist	reduces ATP binding to inhibit a wide range of kinases	rheumatoid arthritis, immune thrombocytopenic purpura
*GRIK5*	glutamate receptor ionotropic, kainate 5	Q16478	Glutamic acid	DB00142	small molecule	approved, nutraceutical	agonist	activates both ionotropic and metabotropic glutamate receptors	nutritional supplement
			Butabarbital	DB00237	small molecule	approved	antagonist	potentiates GABAergic neurons while inhibiting neuronal acetylcholine and glutamate receptors	sedative
*GRIN2B*	glutamate receptor ionotropic, NMDA 2B	Q13224	Acetylcysteine	DB06151	small molecule	approved, investigational	agonist	cysteine/glutamate transporter activator	mucolytic therapy and management of acetaminophen overdose
			Felbamate^a^	DB00949	small molecule	approved	antagonist	antagonist at the strychnine-insensitive glycine-recognition site of the N-methyl-D-aspartate (NMDA) receptor-ionophore complex	anticonvulsant
*GSK3A*	glycogen synthase kinase-3 alpha	P49840	Fostamatinib	DB12010	small molecule	approved, investigational	antagonist	antagonizes GSK3A that is a negative regulator in the hormonal control of glucose homeostasis, Wnt signaling and regulation of transcription factors, and microtubules	rheumatoid arthritis, immune thrombocytopenic purpura
*HCAR2*	hydroxycarboxylic acid receptor 2	Q8TDS4	Niacin^a^	DB00627	small molecule	approved, investigational, nutraceutical	agonist	vitamin B3, mediates increased adiponectin secretion and decreased lipolysis through G(i)-protein-mediated inhibition of adenylyl cyclase that may affect activity of cAMP-dependent phosphorylation of target proteins, leading to neutrophil apoptosis	dietary supplementation; niacin is a B vitamin used to treat vitamin deficiencies as well as hyperlipidemia, dyslipidemia, hypertriglyceridemia, and for anti-atherosclerotic activity, potential neuroimmune effects; investigational in osteoarthritis
*HCRTR2*^c^	orexin receptor type 2	O43614	Suvorexant^a^	DB09034	small molecule	approved, investigational	antagonist	antagonist of orexin receptors OX1R and OX2R that promotes sleep	insomnia
*HDAC3*^b^	histone deacetylase 3	O15379	Vorinostat^a^	DB02546	small molecule	approved, investigational	antagonist	inhibits enzyme activity of class I and class II histone deacetylases	cutaneous T cell lymphoma
*HDAC7*^b^	histone deacetylase 7	Q8WUI4	Panobinostat^a^	DB06603	small molecule	approved, investigational	antagonist	selectively inhibits the class I (HDAC1, HDAC2, HDAC3, and HDAC8), II (HDAC4, HDAC5, HDAC6, HDAC7, HDAC9, and HDAC10), and IV (HDAC11) mammalian histone deacetylase families of enzymes, protein metabolism inhibitor, cell-cycle inhibitor	refractory multiple myeloma
*HDAC9*	histone deacetylase 9	Q9UKVO	Valproic acid	DB00313	small molecule	approved, investigational	antagonist	direct histone deactylase (HDAC) inhibitor	anticonvulsant, migraine, mania associated with bipolar disorder
*ICAM1*	intercellular adhesion molecule 1	P05362	Nafamostat	DB12598	small molecule	investigational	antagonist	inhibits several enzyme systems, including coagulation and fibrinolytic systems (thrombin, Xa, and XIIa), kallikrein-kinin system, complement system, pancreatic proteases, and activation of protease-activated receptors; inhibits lipopolysaccharide-induced nitric oxide production, apoptosis, and interleukin (IL)-6 and IL-8 levels in cultured human trophoblasts; antioxidant in TNF-α-induced ROS production	anticoagulant
*IGF1R*^b^^,^^c^	insulin-like growth factor 1 receptor	P08069	Mecasermin^a^	DB01277	protein	approved	agonist	mediates effects of growth hormone through cell surface receptor tyrosine kinases, anabolic	growth failure in children due to IGF1 deficiency, experimental in osteoarthritis
			Teprotumumab^a^	DB06343	monoclonal antibody	approved, investigational	antagonist	fully human IgG1 monoclonal antibody directed against the human insulin-like growth factor-1 receptor, inhibits downstream effects of IGF1R signaling	thyroid eye disease
*JAK2*	tyrosine-protein kinase JAK2	O60674	Baricitinib^a^	DB11817	small molecule	approved, investigational	antagonist	selective and reversible Janus kinase 1 (JAK1) and 2 (JAK2) inhibitor	rheumatoid arthritis resistant to non-biologic disease-modifying anti-rheumatic drugs, treatment of COVID-19
*KCNH2*^b^	potassium voltage-gated channel subfamily H member 2	Q12809	Sotalol^a^	DB00489	small molecule	approved	antagonist	inhibits beta-1 adrenoceptors in the myocardium and competitive inhibitor of rapid potassium channels to slow repolarization, lengthen the QT interval, and slow and shorten conduction of action potentials through the atria	atrial and ventricular arrhythmias
*LAMC2*	laminin subunit gamma-1	P11047	Lanoteplase	DB06245	protein	investigational	unknown	third generation recombinant plasminogen activator; serine protease that binds to fibrin in thrombus and converts plasminogen to plasmin to degrade fibrin clot	investigational for treatment of myocardial infarction
*MAP2K1*^c^	mitogen-activated protein kinase kinase 1	Q02750	Binimetinib^a^	DB11967	small molecule	approved, investigational	antagonist	potent and selective oral mitogen-activated protein kinase 1/2 (MEK 1/2) inhibitor	metastatic melanoma, investigational in rheumatoid arthritis
*MAP2K6*^c^	dual specificity mitogen-activated protein kinase kinase 6	P52564	Fostamatinib	DB12010	small molecule	approved, investigational	antagonist	tyrosine kinase inhibitor, role in osteoclast activation and endochondral ossification through SOX9	rheumatoid arthritis, immune thrombocytopenic purpura
*MAPK14*^b^	mitogen-activated protein kinase 14	Q16539	PH-797804	DB07835	small molecule	investigational	antagonist	investigated for the treatment of osteoarthritis.	
*NFKB1*	nuclear factor kappa beta p105 subunit	P19838	Thalidomide^a^	DB01041	small molecule	approved, withdrawn for hypnotic indications	agonist	modulates cytokine release, catalyzes sulfation of chondroitin	immunosuppressive, anti-angiogenic
*NISCH*	nischarin	Q9Y2I1	Tizanidine^a^	DB00697	small molecule	approved	agonist	alpha-2 adrenergic receptor agonist causing presynaptic inhibition of motor neurons	muscle spasm
			Tepotinib	DB15133	small molecule	approved, investigational	antagonist	mesenchymal-epithelial transition factor tyrosine kinase inhibitor	metastatic non-small cell lung cancer
*NOS3*^b^^,^^c^	nitric oxide synthetase	P29474	Levamlodipine^a^	DB09237	small molecule	approved, investigational	agonist	inhibits L-type calcium channels in vascular smooth muscle, reducing peripheral vascular resistance	hypertension, including intracranial
			Miconazole	DB01110	small molecule	approved, investigational	antagonist	broad-spectrum azole antifungal with inhibitory action on endogenous and inducible nitric oxide synthetase in humans	fungal infections
*NR3C1*^b^^,^^c^	glucocorticoid receptor	P04150	Prednisolone^a^	DB00860	small molecule	approved	agonist	multiple anti-inflammatory, immunosuppressive, anti-neoplastic, and vasoconstrictive actions	multiple indications involving inflammation and immunity, investigational in osteoarthritis
			Budesonide	DB01222	small molecule	approved	antagonist		inflammatory bowel disease, chronic inflammatory lung conditions
*PAK1*	serine/threonine protein kinase 1	Q13153	Fostamatinib	DB12010	small molecule	approved, investigational	antagonist	tyrosine kinase inhibitor, role in osteoclast activation and endochondral ossification through SOX9	rheumatoid arthritis, immune thrombocytopenic purpura
*PPARD*^b^	peroxisome proliferator-activated receptor delta	Q03181	Treprostinil^a^	DB00374	small molecule	approved	agonist	synthetic prostacyclin analog, vasodilatation, anti-platelet	pulmonary artery hypertension
			Sulindac	DB00605	small molecule	approved, investigational	antagonist	negative regulator of PPARD, non-steroidal anti-inflammatory agent	symptom management in osteoarthritis and inflammatory joint conditions
*PPARG*^b^	peroxisome proliferator-activated receptor gamma	P37231	Rosiglitazone^a^	DB00412	small molecule	approved, investigational	agonist	thiazolidinedione, selective ligand of PPARγ increases insulin sensitivity	diabetes mellitus
*PRKCD*	protein kinase C delta type	Q05655	Ingenol mebutate	DB05013	small molecule	approved	agonist	neutrophil-mediated inflammation, activator of PKC-delta	actinic keratosis
			Fostamatinib	DB12010	small molecule	approved, investigational	antagonist	tyrosine kinase inhibitor, role in osteoclast activation and endochondral ossification through SOX9	rheumatoid arthritis, immune thrombocytopenic purpura
*PTHLH*^c^	parathyroid hormone like hormone	P12272	Teriparatide	DB06285	protein	approved, investigational	agonist	synthetic human parathyroid hormone (PTH) amino acid sequence 1 through 34 of the complete molecule which contains amino acid sequence 1 to 84; endogenous PTH is the primary regulator of calcium and phosphate metabolism in bone and kidney	osteoporosis, investigational in osteoarthritis
*RAF1*^b^	RAF proto-oncogene serine/threonine-protein kinase	P04049	Cholecystokinin	DB08862	small molecule	approved, investigational	agonist	peptide hormone synthesized in the human gut, also the most ubiquitously found neuropeptide in the human brain; regulates gallbladder contraction, intestinal motility, and pancreatic enzyme secretion and growth	pancreatic insufficiency and diagnostic for gallbladder disorders
			Sorafenib^a^	DB00398	small molecule	approved, investigational	antagonist	interacts with multiple intracellular (CRAF, BRAF, and mutant BRAF) and cell surface kinases (KIT, FLT-3, VEGFR-2, VEGFR-3, and PDGFR-β) that are involved in angiogenesis, thus sorafenib reduces blood flow to the tumor; Sorafenib is targeting the Raf/Mek/Erk pathway; by inhibiting these kinases, genetic transcription involving cell proliferation and angiogenesis is inhibited	advanced renal cell carcinoma and hepatocellular carcinoma
*S1PR5*	sphingosine 1-phosphate receptor 5	Q9H228	Fingolimod^a^	DB08868	small molecule	approved, investigational	antagonist	sphingosine 1-phosphate-induced cell proliferation, survival, and transcriptional activation	multiple sclerosis
*SLC1A1*	excitatory amino acid transporter 3	P43005	Pregabalin^a^	DB00230	small molecule	approved, investigational	agonist	structurally similar to gamma-aminobutyric acid (GABA)—an inhibitory neurotransmitter	neuropathic pain, and in chronic pain in arthritis
*SLC6A1*^b^	sodium- and chloride-dependent GABA transporter 1	P30531	Tiagabine^a^	DB00906	small molecule	approved, investigational	antagonist	selective gamma amino butyric acid (GABA) reuptake inhibitor. Inhibits GABA reuptake into presynaptic neurons	anticonvulsant, treatment of panic disorder
*SMO*^c^	smoothened frizzled family receptor	Q99835	Fluocinonide	DB01047	small molecule	approved, investigational	agonist	glucocorticoid steroid with Wnt-protein binding SMO agonist activity	Inflammatory and pruritic manifestations of corticosteroid-responsive dermatoses
			Vismodegib^a^	DB08828	small molecule	approved, investigational	antagonist	inhibits *IHH* signaling	basal cell carcinoma
*SST*^b^	somatostatin		Somatostatin	DB09099	protein	approved, investigational	agonist	peptide hormone that regulates the endocrine system and affects neurotransmission and cell proliferation via interaction with G protein-coupled somatostatin receptors and inhibition of the release of numerous secondary hormones	multiple endocrine indications, including carcinoid syndrome
*TGFB1*^c^	transforming growth factor beta 1	P01137	Terazocin	DB1162	small molecule	approved	agonist	multifunctional peptide: cell growth, proliferation and differentiation	benign prostatic hyperplasia, hypertension
			Hyaluronidase	DB14740	protein	approved	antagonist	cleaves hyaluronic acid at the glucosaminidic bond between C1 of glucosamine and C4 of glucuronic acid	increases dispersion of subcutaneously injected fluids and drugs
*TNFSF11*^b^^,^^c^	tumor necrosis factor ligand superfamily member 11	O14788	Denosumab	DB06643	monoclonal antibody	approved, investigational	antagonist	inhibits osteoclast formation, function, and survival	osteoporosis, bone metastasis, investigational in osteoarthritis
*TPO*^b^	thyroid peroxidase	P07202	Carbimazole^a^	DB00389	small molecule	approved, investigational	antagonist	an imidazole anti-thyroid agent; Carbimazole is metabolized to methimazole, which is responsible for the anti-thyroid activity	hyperthyroidism
*TYK2*	non-receptor tyrosine-protein kinase TYK2	P29597	Tofacitinib^a^	DB08895	small molecule	approved, investigational	antagonist	Janus kinase inhibitor	rheumatoid arthritis resistant to non-biologic disease-modifying anti-rheumatic drugs
*VDR*^b^^,^^c^	vitamin D receptor	P11473	Calcitriol^a^	DB00136	small molecule	approved	agonist	active metabolite of vitamin D	vitamin D deficiency, chronic kidney disease, hyperparathyroidism (secondary), investigational in osteoarthritis
			Paracalcitol^a^	DB00910	small molecule	approved, investigational	antagonist	synthetic vitamin D analog	secondary hyperparathyroidism associated with chronic renal failure

Genes are identified according to the Ensembl GeneName for the gene. Both agonists and antagonists of the target protein are shown. DrugBank information on the tier 1 likely effector genes.

a Indicates that multiple drugs with similar mechanisms of action are identified for a given target. Here, an example drug from the class is shown to represent an identified mechanism of action on the target-encoded protein.

b Denotes associated with newly reported signal.

c Denotes effector genes with at least 3 lines of evidence.

Within tier 1, ten candidates have previously been studied in clinical trials of efficacy or in cohort studies of osteoarthritis (six arising from new signals: *PPARD*, *NR3C1*, *VDR*, *MAPK14*, *IGF1R*, and *CHST3*). The *PPARD* antagonist sulindac has marketing authorization as a non-steroidal anti-inflammatory drug (NSAID) in osteoarthritis for its prostaglandin synthase activity. The *SLC1A1* agonist and neuropathic pain inhibitor pregabalin is commonly prescribed in osteoarthritis. Pregabalin has some supportive clinical trial data for its co-prescription with the NSAID meloxicam in the short-term treatment of pain in knee osteoarthritis (Ohtori et al., 2013). *NR3C1* encodes the glucocorticoid receptor, the activation of which has broad anti-inflammatory and immunomodulatory actions with marketing authorization for several agonist molecules. One of these, prednisolone, has long been used as a disease modifying agent in inflammatory arthritis and in the recent Heart Outcomes Prevention Evaluation (HOPE) study was found to be effective in reducing pain and synovitis in hand osteoarthritis (Kroon et al., 2019). Cathepsin K (encoded by the *CTSK* gene) is an enzyme that plays a critical role in collagen degradation within osteoclasts, and MIV-711 is a selective cathepsin K inhibitor that has recently been shown in a phase 2 clinical trial to be effective in reducing structural damage in patients with knee osteoarthritis (Conaghan et al., 2020). *VDR* encodes the vitamin D receptor, the activation of which is a major regulator of calcium metabolism. The results of clinical trials of vitamin D supplementation on symptoms and structural damage in knee osteoarthritis have been mixed (Arden et al., 2016; Jin et al., 2016; McAlindon et al., 2013; Sanghi et al., 2013; Zheng et al., 2017) but may suggest a small benefit in patients with vitamin D deficiency. *EGLN2* encodes Egl nine homolog 2, a prolyl hydroxylase that mediates hydroxylation of proline and thus contributes to collagen and proteoglycan synthesis. Supplementation of its agonist, ascorbic acid (vitamin C), has been associated with joint health in observational cohorts, although with mixed effects (Joseph et al., 2020; McAlindon et al., 1996; Peregoy and Wilder, 2011). Deficiency of the *HCAR2* agonist niacin (vitamin B3) was associated with knee osteoarthritis progression in the Japanese ROAD cohort (Muraki et al., 2014). The MAPK14 antagonist PH-797804 has been studied in a phase 2 clinical trial to examine the pain relief of PH-797804 alone or with naproxen in subjects with osteoarthritis of the knee (NCT01102660), although we are not aware of any trial results reporting in PubMed or on ClinicalTrials.gov. Finally, the carbohydrate sulfotransferase 3 agonist thalidomide has been shown to attenuate early osteoarthritis development in a mouse medial meniscus destabilization model through a mechanism involving the downregulation of vascular endothelial growth factor (VEGF) expression (Seegmiller et al., 2019).

All of the 45 further tier 1 druggable targets have market authorization or are in clinical development for other indications (Table 4). Ten of these are high-confidence effector genes and 16 arise from new genetic signals. The functional and epidemiological evidence of their roles in clinical osteoarthritis presented here provides support for early repurposing investigation. One antibody small molecule, fostamatinib, appears multiple times in Table 4 as a tyrosine kinase inhibitor that targets *AAK1*, *EPHA5*, *GAK*, *GSK3A*, *MAP2K1*, *MAP2K6*, *PAK1*, and *PRKCD*, and has marketing approval as a biologic disease-modifying anti-rheumatic drug (DMARD). The *JAK2* antibody baricitinib and the *TYK2* antibody tofacitinib are both marketed as biologic DMARDs, and the *MAP2K1* antibody binimetinib is currently in phase 3 clinical trials as a biologic DMARD. Each of these drugs therefore present a clinical opportunity and putative mechanism for repurposing studies in osteoarthritis. Of the remaining tier 1 and tier 2 druggable targets, the potentially actionable molecules are at an earlier stage of development and present a more distant repurposing opportunity.

## Discussion

Our findings have generated further knowledge on the differences between weight bearing and non-weight bearing joints and point to mechanisms that are common to disease development at any joint, and joint-type-specific. Indeed, bone and cartilage development pathways were enriched in signals traversing weight bearing and non-weight bearing joints, identifying joint development as a common mechanism for any form of osteoarthritis (Table S13).

We have been able to establish molecular links between the disease and its main symptom, pain. We demonstrate genetic correlation between osteoarthritis and pain-related phenotypes and identify signal enrichment in neurological pathways (Table S13). Furthermore, several of the high-confidence effector genes have a role in neuropathology. The majority of osteoarthritis cases in this study were defined as total joint replacement and/or self-reported osteoarthritis, and both of these disease phenotypes are highly driven by pain. Identification of these genes can also have implications for further joint pain-related disorders, for which insights have been limited to date.

A large number of the high-confidence effector genes converge on the endochondral pathway, playing an essential role in homeostasis of the chondrocyte (Figure 3) and osteophytosis. Several of the identified genes are important in TGF-β signaling and function. The newly identified fibrillin 2 (*FNB2*) signal, together with *LTBP1* and *LTBP3*, regulate the availability of active TGFB1. TGFB1 is the major form of TGFB in cartilage and can activate a cascade of downstream genes through SMAD3-signaling, including ECM-genes which have been identified in our current study, such as carbohydrate sulfotransferase 3 (*CHST3*) (Zhou et al., 2020).

Our data provide evidence for the FGF-signaling cascade (*FGFR3*, *FGF18*, and *PIK3R1*) being causally involved in osteoarthritis (Figure 3). FGF18 is currently being tested in clinical trials for its effectiveness in osteoarthritis (Hochberg et al., 2019). The newly identified molecular player phosphoinositide-3-kinase regulatory subunit 1 (*PIK3R1*) encodes the p85a, p55a, and p50a regulatory subunits of class IA phosphatidylinositol 3 kinases (PI3Ks), which are known to play a key role in the metabolic actions of insulin and are required for adipogenesis (Kim et al., 2014; Thauvin-Robinet et al., 2013). Mutations in *PIK3R1* cause agammaglobulinemia 7 (Conley et al., 2012), immunodeficiency 36 (Deau et al., 2014; Lucas et al., 2014), and SHORT syndrome (Dyment et al., 2013), which is characterized by short stature, hyperextensibility of joints, ocular depression, Rieger anomaly, and teething delay (Dyment et al., 2013; Thauvin-Robinet et al., 2013). The balance between chondrocyte proliferation, differentiation, and hypertrophic conversion is controlled by crosstalk between several signaling pathways, of which we find causal evidence here: PTHLH and IHH-signaling (*SMO* and *PTCH1*) antagonize signaling through FGFR3. In addition, we identify two independent genetic variants implicating noggin (*NOG*) as an osteoarthritis effector gene. Noggin binds to TGFB, BMPs, and GDF5 and thereby prevents binding to the cognate receptor. Mutations in *NOG* cause a whole range of bone and cartilage phenotype depending on the severity of the mutation (Lehmann et al., 2007).

Several of the putative osteoarthritis causal genes are involved in developmental pathways (Figure 3). Skeletal development can be linked to osteoarthritis in several ways. First, skeletal developmental genes are involved in joint (tissue) characteristics before onset of disease such as cartilage thickness (*TGFA*, *FGFR3*, *RUNX2*, and *PIK3R1*) (Castaño-Betancourt et al., 2016) or joint shape (resulting in different loading of the joint). Second, the skeletal development pathway could be involved in the reaction to damage in the joint. Depending on the specific genetic makeup, each individual reaction to a damaging trigger to the joint could be different, thereby determining the risk of developing osteoarthritis upon trauma or mechanical overload. Pathway analysis performed on the current study signals further corroborated this, because it revealed evidence for enrichment of pathways typically involved in reaction to damage.

Our data also suggest that subtle changes in pivotal osteochondrogenic pathways lead to an adverse response to joint damage and/or overload. This may catalyze a fibrotic response both in cartilage and in the synovium. We identified tenascin C (*TNC*) as one of the high-confidence effector genes (Fu et al., 2017; Imanaka-Yoshida et al., 2020) (Figure 3). TNC is a component of the extracellular matrix and is involved with organ fibrosis, inflammation, and cardiovascular disease (Golledge et al., 2011; Yasuda et al., 2018). The formation of fibrocartilage and fibrosis in the joint is a major contributor to the degenerative changes in osteoarthritis (Rim and Ju, 2020). Further, elevated levels of TGF-β signaling are associated with the pathological and fibrosis changes (van der Kraan, 2017). TGF-β is also a potent inducer of epithelial-mesenchymal transition (EMT) (Nieto et al., 2016; Stone et al., 2016). EMT, a process whereby fully differentiated epithelial cells undergo transition to a mesenchymal phenotype giving rise to fibroblasts, is a driver of early fibrosis, which is a typical response to injury or pathological changes and inflammation, all common endpoint outcomes in osteoarthritis. The severity of fibrosis contributes to the degree of degenerative changes that lead to pain in osteoarthritis. We have identified significant association of variants in many of the genes involved in the induction (e.g., EMT genes, *CUX1* and multiple molecular components of the TGF-β pathway) and progression of fibrosis (ECM genes e.g., *TNC*, TGF-β signaling *FNB2*, *LTBP1*, *LTBP3*, *TGFB1*, and *SMAD3*) (Figure 3B). These findings indicate that combined variation in the regulation of these genes may collectively contribute to the susceptibility and severity of degenerative changes in osteoarthritis.

Seventy-one of the implicated genes code for molecules that are the targets of approved (licensed) drugs and drugs in clinical development. Our findings substantially strengthen the evidence for these potential therapeutics, provide drug repositioning opportunities, and offer a solid basis on which to develop, or repurpose, such interventions for osteoarthritis.

Our work provides a robust springboard for follow-up functional and clinical studies. We have demonstrated clear differences between distinct osteoarthritis patient populations, for example based on disease severity, joint site affected, and sex. We enhance our understanding of the genetic etiology of disease, shed biological insights, and provide a stepping stone for translating genetic associations into osteoarthritis drug development, ultimately helping to catalyze an improvement in the lives of patients suffering from osteoarthritis.

### Limitations of the study

Enhancing population diversity in genetic association studies is important for discovering risk variants, pinpointing likely causal alleles, improving risk prediction, and ensuring the transferability of findings across global populations. In this work, less than 3% of contributing subjects were of non-European ancestry. Going forward, the identification and inclusion of diverse populations in osteoarthritis genetic association studies is urgently needed.

Disentangling mechanisms that are active at the point of disease initiation versus those activated during the natural history of disease warrant animal model studies in which disease dynamics can be studied in depth. Indeed, investment in mechanistic studies of the newly identified high-value targets will be important next steps. Clinical trials of intervention will be needed to take our findings forward into mechanism and clinical outcome, therefore elucidating how to target the implicated genes and proteins, how downstream events will be affected, and, ultimately, how these interventions will affect disease outcome in the patient.

## STAR★Methods

### Key resources table

**Table:** 

REAGENT or RESOURCE	SOURCE	IDENTIFIER
**Deposited data**		

Genome-wide summary statistics for all GO meta-analysis results generated.	This paper.	Available from the ‘Downloads’ page of the Musculoskeletal Knowledge Portal (https://mskkp.org).
Cohort-level genome-wide summary statistics for TwinsUK are available on request.	This paper. Refer to den Hollander et al., 2017 for additional cohort genetic information.	Contact Eleftheria Zeggini (eleftheria.zeggini@helmholtz-muenchen.de).
Cohort-level raw data for TwinsUK are available by application.	den Hollander et al., 2017.	https://twinsuk.ac.uk/resources-for-researchers/access-our-data/
Cohort-level genome-wide summary statistics for Japan Study is available on request. Raw data are not available due to patient confidentiality or consent restrictions or ethical and legal restrictions.	This paper. Refer to Nakajima et al., 2010 for additional cohort genetic information.	Contact Shiro Kegawa (sikegawa@ims.u-tokyo.ac.jp).
Cohort-level genome-wide summary statistics for Nurses’ Health Studies are available on request.	This paper.	Contact Eleftheria Zeggini (eleftheria.zeggini@helmholtz-muenchen.de).
Cohort-level raw data for Nurses’ Health Studies are available upon application.	PIs: Peter Kraft & Jae Hee Kang.	https://www.nurseshealthstudy.org/researchers. Contact email: nhsaccess@channing.harvard.edu and https://sites.sph.harvard.edu/hpfs/for-collaborators/
Cohort-level genome-wide summary statistics for deCODE are available on request. Raw data are not available due to patient confidentiality or consent restrictions or ethical and legal restrictions.	This paper. Refer to Styrkarsdottir et al., 2018 for additional cohort genetic information.	Contact https://www.decode.com
Cohort-level genome-wide summary statistics for Geisinger are available by application. Raw data are not available due to patient confidentiality or consent restrictions or ethical and legal restrictions.	This paper. Refer to Zhang et al., 2021 for additional cohort genetic information.	Summary statistics are available for only academic organizations upon approval by Geisinger and Regeneron. Data use agreement is required. https://www.geisinger.org/precision-health/mycode/discovehr-project
Cohort-level genome-wide summary statistics for Rotterdam Study Cohorts are available on request.	This paper. Refer to Ikram et al., 2020 for additional cohort genetic information.	Contact Joyce van Meurs (j.vanmeurs@erasmusmc.nl).
Due to ethical and legal restrictions, individual-level data/raw data of the Rotterdam Study cannot be made publicly available. Data are available upon request and subject to local rules and regulations. This includes submitting a proposal to the management team of RS, where upon approval, analysis needs to be done on a local server with protected access, complying with GDPR regulations.	This paper. Refer to Ikram et al., 2020 for additional cohort genetic information.	Application to the data manager of the Rotterdam Study Frank van Rooij (f.vanrooij@erasmusmc.nl).
Cohort-level summary statistics for HK Spine OA are available on application. Raw data are not available due to patient confidentiality or consent restrictions or ethical and legal restrictions.	This paper. Refer to Li et al., 2016 for additional genetic information.	Available upon application to Kathryn Cheah (kathycheah@hku.hk).
Cohort-level summary statistics and raw data for HUNT are available upon application.	This paper.	Available upon application to kontakt@hunt.ntnu.no
Cohort-level summary statistics and raw data for Estonian Biobank (EGCUT) are available upon application.	This paper.	Available upon application to Maris Teder-Laving (maris.teder-laving@ut.ee) or https://genomics.ut.ee/et
Cohort-level summary statistics For GARP study and LLS are available upon request. Raw data are not available due to patient confidentiality or consent restrictions or ethical and legal restrictions.	This paper. Refer to the following for additional genetic information. GARP study: Meulenbelt et al., 2008; https://www.lumc.nl/org/reumatologie/research/artrose/9031609354853/?setlanguage=English&setcountry=en LLS: Beekman et al., 2010; https://www.leidenlangleven.nl/en/home	Contact Ingrid Meulenbelt (i.meulenbelt@lumc.nl).
Cohort-level summary statistics for UK Biobank are available by request.	This paper. Refer to Bycroft et al., 2018 for additional cohort genetic information.	Contact Eleftheria Zeggini (eleftheria.zeggini@helmholtz-muenchen.de).
Raw data for UK Biobank are available by application.	Bycroft et al., 2018.	https://www.ukbiobank.ac.uk/enable-your-research/apply-for-access
Cohort-level summary statistics for arcOGEN and UKHLS are available by request.	This paper.	Contact Eleftheria Zeggini (eleftheria.zeggini@helmholtz-muenchen.de).
Raw data for arcOGEN are available from the European Genome-phenome Archive (EGA).	Zeggini et al., 2012.	EGAS00001001017.
Raw data for UKHLS are available by application.	University of Essex, Institute for Social and Economic Research, NatCen Social Research, Kantar Public. (2020). Understanding Society: Waves 1-10, 2009-2019 and Harmonised BHPS: Waves 1-18, 1991-2009. [data collection]. 13th Edition. UK Data Service. SN: 6614, https://doi.org/10.5255/UKDA-SN-6614-14.	https://www.understandingsociety.ac.uk/
Cohort-level summary statistics for ARGO-Larissa are available by request.	This paper.	Contact Eleftheria Zeggini (eleftheria.zeggini@helmholtz-muenchen.de).
Raw data for ARGO-Larissa are available from the European Genome-phenome Archive (EGA).	This paper.	EGAS00001000917.
Cohort-level summary statistics and raw data for ARGO-Athens are available by request.	This paper.	Contact Eleftheria Zeggini (eleftheria.zeggini@helmholtz-muenchen.de).
Resource related to LD SCore analysis: Pre-computed LD scores for European populations.	Bulik-Sullivan et al., 2015a, 2015b	https://alkesgroup.broadinstitute.org/LDSCORE/eur_w_ld_chr.tar.bz2
Resource related to drug identification analysis: DrugBank database.	Wishart et al., 2018	https://go.drugbank.com
Resource related to eQTL colocalization, causal inference and tissue specificity analyses: GTEx.	Battle et al., 2017	https://www.gtexportal.org/
Resource related to meta-analyses and tissue specificity analysis: 1000 Genomes Project.	Auton et al., 2015	http://www.internationalgenome.org/
Resource related to tissue specificity analysis: ROADMAP.	Kundaje et al., 2015	http://www.roadmapepigenomics.org/
Used as a reference panel of genotype imputation and allele frequency checking in meta-analyses: The Haplotype Reference Consortium (HRC).	McCarthy et al., 2016	http://www.haplotype-reference-consortium.org/
Human Phenotype Ontology (HPO) database was used to identify if any of the high confidence effector genes are implicated in monogenic and rare human diseases.	Köhler et al., 2021	https://hpo.jax.org/
MGI Mouse Genome Informatics database was used to extract all mouse knockout phenotypes.	Bult et al., 2019; Finger et al., 2015	http://www.informatics.jax.org/
OMIM database resource was used to identify human genes linked to monogenetic pain disorders.	Amberger et al., 2015	https://www.omim.org/
Human Pain Genetics Database resource was used to identify genes linked to pain.	Meloto et al., 2018	https://humanpaingenetics.org/hpgdb/
Orphanet database resource was used to identify if any of the high confidence effector genes are implicated in monogenic and rare human diseases.	Orphanet: an online database of rare diseases and orphan drugs. Copyright, INSERM 1997.	https://www.orpha.net/consor/cgi-bin/index.php?lng=EN
Ensembl resource was used to obtain genes and variants annotation.	Yates et al., 2015, 2020	http://www.ensembl.org//useast.ensembl.org/index.html?redirectsrc=//www.ensembl.org%2Findex.html
Resource related to tissue specificity analysis: ENCODE.	Snyder et al., 2020	https://www.encodeproject.org
Resource related to drug identification analysis: ChEMBL database.	Gaulton et al., 2017	https://www.ebi.ac.uk/chembl/
DECIPHER database resource was used to identify if any of the high confidence effector genes are implicated in monogenic and rare human diseases.	Firth et al., 2009	https://www.deciphergenomics.org/about/overview

**Software and algorithms**		

R statistical software	R Project for Statistical Computing.	https://www.R-project.org/
EasyQC	Winkler et al., 2014	https://www.uni-regensburg.de/medizin/epidemiologie-praeventivmedizin/genetische-epidemiologie/software/
METAL	Willer et al., 2010	https://genome.sph.umich.edu/wiki/METAL_Documentation
GWAMA	Mägi et al., 2010; Mägi and Morris, 2010	https://bmcbioinformatics.biomedcentral.com/articles/10.1186/1471-2105-11-288
PLINK 1.9	Purcell et al., 2007	https://www.cog-genomics.org/plink/1.9/
COJO in GCTA	Yang et al., 2011, 2012	https://cnsgenomics.com/software/gcta/#COJO
FUMA	Watanabe et al., 2017	https://fuma.ctglab.nl
LDHub	Zheng et al., 2017	https://github.com/bulik/ldsc
PRsice2	Choi and O’Reilly, 2019; Choi et al., 2020	https://www.prsice.info
LDpred	Vilhjálmsson et al., 2015	https://github.com/bvilhjal/ldpred
LDSC (LD SCore)	Bulik-Sullivan et al., 2015a, 2015b	https://github.com/bulik/ldsc/
fast.coloc	Genetics ToolboX Created by Toby Johnson 2019.	https://github.com/tobyjohnson/gtx/blob/526120435bb3e29c39fc71604eee03a371ec3753/R/coloc.R
ConsensusPathDB-human	Kamburov et al., 2011	http://cpdb.molgen.mpg.de/
PhenoScannerV2	Kamat et al., 2019	http://www.phenoscanner.medschl.cam.ac.uk/
Adobe illustrator	Vector graphics editor and design program developed and marketed by Adobe	https://www.adobe.com/de/creativecloud/illustration.html
Custom scripts for quality control and analyses.	This paper.	https://doi.org/10.5281/zenodo.5036143

**Other**		

Website for GO Consortium.	This paper.	https://www.genetics-osteoarthritis.com/
Website for Avon Longitudinal Study of Parents and Children (ALSPAC).	Boyd et al., 2013; Fraser et al., 2013	http://www.bristol.ac.uk/alspac/
Website for Understanding Society study.	University of Essex, Institute for Social and Economic Research, NatCen Social Research, Kantar Public. (2020). Understanding Society: Waves 1-10, 2009-2019 and Harmonised BHPS: Waves 1-18, 1991-2009. [data collection]. 13th Edition. UK Data Service. SN: 6614, https://doi.org/10.5255/UKDA-SN-6614-14.	https://www.understandingsociety.ac.uk/
Website of UK Biobank.	Bycroft et al., 2018	https://www.ukbiobank.ac.uk/

### Resource availability

#### Lead contact

Further information requests should be directed to the lead contact, Eleftheria Zeggini (eleftheria.zeggini@helmholtz-muenchen.de).

#### Materials availability

This study did not generate new unique reagents.

### Experimental model and subject details

#### Study cohorts

Detailed information relating to the human subjects for each cohort, is provided in Table S1. This includes the number of individuals in each cohort, sex information and ethnicity.

##### arcOGEN

Arthritis Research UK Osteoarthritis Genetics (arcOGEN) is a collection of 7410 unrelated, UK-based individuals of European ancestry with knee and/or hip osteoarthritis from the arcOGEN Consortium (Zeggini et al., 2012). Samples were collected in 2 stages from 10 United Kingdom locations (London, Nottingham, Oxford, Sheffield, Southampton, Edinburgh, Newcastle-Upon-Tyne, Sheffield, Wansbeck, and Worcester). The majority of cases had primary OA requiring joint replacement of the hip or knee while a smaller number were ascertained by radiographic evidence of disease (Kellgren-Lawrence (KL) grade ≥ 2). The exclusion criteria included the need for joint replacement due to fracture, secondary OA of any cause, and developmental, vascular, or infective causes of joint disease.

##### United Kingdom Household Longitudinal Study (UKHLS)

UKHLS also known as Understanding Society, is a longitudinal panel survey of 40,000 UK households (England, Scotland, Wales and Northern Ireland) representative of the UK population. Participants are surveyed annually since 2009 and contribute information relating to their socioeconomic circumstances, attitudes, and behaviors via a computer assisted interview. The study includes phenotypical data for a representative sample of participants for a wide range of social and economic indicators as well as a biological sample collection encompassing biometric, physiological, biochemical, and hematological measurements and self-reported medical history and medication use. https://www.understandingsociety.ac.uk/.

##### ARGO-Larissa

The ARGO-Larissa study was set up to investigate the genetic architecture of knee OA in a Greek population. It included individuals with primary knee OA undergoing total knee arthroplasty. The osteoarthritis participants’ recruitment was conducted in the city of Larissa, central Greece.

##### ARGO-Athens

The ARGO study, was set up to investigate the genetic architecture of hip and knee OA in a Greek population. More than 1,500 patients with severe OA, undergoing hip and/or knee total joint replacement were recruited from the cities of Athens and Larissa. The ARGO collection was conducted in three public hospitals (Attikon University General Hospital of Athens, Nea Ionia General Hospital Konstantopouleio, and KAT Hospital) and one private hospital (Lefkos Stavros General Hospital) in the city of Athens, Greece between February of 2015 and March 2017.

##### UK Biobank

The UK Biobank study is a large population-based prospective study of > 500,000 participants with ages ranging 40–69 years. In total, 503,325 participants who registered in the National Health Service were recruited out of 9.2 million mailed invitations between 2006 and 2010 in 22 assessment centers throughout the UK (Sudlow et al., 2015). Most participants visited the center once, but some individuals visited the center at up to three times. Baseline data were collected using electronic signed consent, a self-completed touch-screen questionnaire, a brief computer-assisted interview, physical and functional measures, and collection of biological samples and genetic data. The UK Biobank genetic data contains genotypes for 488,377 participants. All detailed genotyping, quality control, and imputation procedures are described at the UK Biobank website (https://biobank.ctsu.ox.ac.uk). Briefly, 50,000 samples were genotyped using the UKBiLEVE array and the remaining samples were genotyped using the UK Biobank Axiom array (Affymetrix) for ∼800,000 SNPs. Population structure was captured by principal component analysis on ∼500,000 UK Biobank samples using ∼100,000 SNPs. After sample and SNP quality control (QC) of the directly-typed genotypes, resulting in 670,739 autosomal markers in 487,442 individuals, data were prephased using SHAPEIT3 (O’Connell et al., 2016) and imputed using the IMPUTE4 program (https://jmarchini.org/software/). Both analyses were carried out centrally (Bycroft et al., 2018) and the full dataset consisted of approximately 96 million variants in 487,411 individuals. https://www.ukbiobank.ac.uk/

This work was based on the third UK Biobank release, which includes the full set of genotypes imputed on the Haplotype Reference Consortium (McCarthy et al., 2016) and the merged UK10K and 1000 Genomes phase 3 reference panels1000 Genomes Consortium (Huang et al., 2015). Access to UK Biobank genetic and phenotypic data was given through the UK Biobank Resource under application request.

##### Hong Kong work -HKSpineOA

Hong Kong Degenerative Disc Disease Population Cohort (HKDDDPC) is a a population-based cohort with subjects openly recruited via newspapers advertisement, posters and e-mails, regardless of their social and economic status (Li et al., 2016). The study call was for any participant who agreed to a study on the lumbosacral spine with MRI, clinical questionnaires and follow-up assessments. Participants with prior surgical treatment of the spine, spinal tumors and fractures, and marked spinal deformities were excluded from the study. Subjects selected were not based on the presence or absence of clinical symptoms. All qualified subjects underwent T1-weighted axial MRI and T2-weighted sagittal MRI of the lumbosacral spine (L1-S1) after informed consent was obtained from participants and ethics was approved by a local institutional board. MRI Protocol: 1.5T or 3T MRI machines were used for axial and sagittal imaging at L1-S1. Subjects were oriented in the supine position. For T1-weighted axial scans, the field of view was 21cmx21cm, slice thickness was 4mm, slice spacing was 0.4mm, and imaging matrix was 218x256. For T2-weighted sagittal scans, the field of view was 28cmx28cm, slice thickness was 5mm, slice spacing was 1mm, and imaging matrix was 448x336. The repetition time for T1- and T2-weighted MRI were 500ms-800ms and 3320ms respectively, and their echo time was 9.5ms and 85ms. According to the pedicle and disc levels, 11 parallel slices were made at each spinal level with reference to the adjacent endplates. Definition of OA on X-rays: For the lumbar lateral radiographs, each disc level (L1-2, L2-3, L3-4, L4-5, L5-S1) was measured for osteophytes and vertebral narrowing. Grade 0 was considered none; grade 1 was mild; grade 2 was moderate; and grade 3 was severe. The L5-S1 disc was narrowed when its height was less than the disc space of L3-4. Diagnosis of OA was based on the criteria as described by de Schepper et al. (2010). Disc space narrowing was present with grade ≥ 1 and osteophytes with grade ≥ 2. OA had either “narrowing,” “osteophytes” or “both.” Narrowing was considered with grade ≥ 1 narrowing at 2 or more vertebral levels and osteophytes were considered with grade ≥ 2 at 2 or more vertebral levels. With both narrowing and osteophytes, then “both” was considered. Conversion of MRI for OA diagnosis: Conversion of MRI for OA diagnosis was dependent on two MRI ratings: disc bulging and Schneiderman score. Disc bulging was divided into 4 categories: 0 = no disc bulging; 1 = posterior disc bulging (disc displaced beyond a virtual line connecting the posterior edges of two adjacent vertebrae); 2 = disc extrusion (distance between the edge of the protruded disc into the spinal canal was greater than the distance between edges of the base of the disc); 3 = disc sequestration (disc material detached and migrated away from the level of the intervertebral disc) (Cheung et al., 2009; Määttä et al., 2015; Teraguchi et al., 2020). The Schneiderman score (Schneiderman et al., 1987) was used to describe the disc signal intensity and was evaluated by a 4-point scale: 0 = normal disc height and signal intensity; 1 = speckled pattern or heterogeneous decreased disc signal intensity; 2 = diffuse loss of signal; 3 = signal void.

Each lumbar intervertebral disc was rated for disc bulging and Schneidermann score. Lumbar spine OA was defined to be present if (1) At least 1 disc with Schneidermann score 3, OR (2) At least 1 disc with Schneidermann score 2 and Bulging score 2, OR (3) At least 2 discs with Schneidermann score 2 AND disc bulging score 1,

There were 587 of subjects with X-rays as well. We validated our definition based on MRI by testing and confirming its equivalence with X-ray diagnoses using these subjects. DNA samples were genotyped using the Illumina humanOmniZhongHua-8 v1.2 BeadChip. Quality control (QC) of the genotyped data were conducted based on pipeline provided by Anderson et al. (2010). Imputation of single nucleotide polymorphisms (SNPs) was performed using reference panels from the Haplotype Reference Consortium (HRC) (McCarthy et al., 2016).

##### The Nord-Trøndelag Health Study (The HUNT Study)

The Nord-Trøndelag Health Study (HUNT) is a large population-based cohort from the county Nord-Trøndelag in Norway. All residents in the county, aged 20 years and older, have been invited to participate. Data was collected through three cross-sectional surveys, HUNT1 (1984-1986), HUNT2 (1995-1997) and HUNT3 (2006-2008), and has been described in detail previously (Krokstad et al., 2013), with the fourth survey recently completed (HUNT4, 2017-2019). DNA from whole blood was collected from HUNT2 and HUNT3, with genotypes available from 71,860 participants. All genotyped participants have signed a written informed consent regarding the use of data from questionnaires, biological samples and linkage to other registries for research purposes. In total, DNA from 71,860 HUNT samples was genotyped using one of three different Illumina HumanCoreExome arrays (HumanCoreExome12 v1.0, HumanCoreExome12 v1.1 and UM HUNT Biobank v1.0). Samples which failed to reach a 99% call rate, had contamination > 2.5% as estimated with BAF Regress (Jun et al., 2012), large chromosomal copy number variants, lower call rate of a technical duplicate pair and twins, gonosomal constellations other than XX and XY, or whose inferred sex contradicted the reported gender, were excluded. Samples that passed quality control were analyzed in a second round of genotype calling following the Genome Studio quality control protocol described elsewhere (Guo et al., 2014). Genomic position, strand orientation and the reference allele of genotyped variants were determined by aligning their probe sequences against the human genome (Genome Reference Consortium Human genome build 37 and revised Cambridge Reference Sequence of the human mitochondrial DNA; http://genome.ucsc.edu) using BLAT (ENCODE Project Consortium, 2012). Variants were excluded if their probe sequences could not be perfectly mapped to the reference genome, cluster separation was < 0.3, Gentrain score was < 0.15, showed deviations from Hardy Weinberg equilibrium in unrelated samples of European ancestry with p value < 0.0001), their call rate was < 99%, or another assay with higher call rate genotyped the same variant. Imputation was performed on the 69,716 samples of recent European ancestry using Minimac3 (v2.0.1, https://genome.sph.umich.edu/wiki/Minimac3) (Das et al., 2016) with default settings (2.5 Mb reference based chunking with 500kb windows) and a customized Haplotype Reference consortium release 1.1 (HRC v1.1) for autosomal variants and HRC v1.1 for chromosome X variants (McCarthy et al., 2016). The customized reference panel represented the merged panel of two reciprocally imputed reference panels: (1) 2,201 low-coverage whole-genome sequences samples from the HUNT study and (2) HRC v1.1 with 1,023 HUNT WGS samples removed before merging. We excluded imputed variants with Rsq < 0.3 resulting in over 24.9 million well-imputed variants.

##### Geisinger

Geisinger is an integrated health care provider located in central and northeastern Pennsylvania and New Jersey. Geisinger’s electronic health record (EHR) consists of comprehensive longitudinal clinical information including patients’ demographic data, diagnoses (including co-morbidities), lab measurements, prescriptions, procedures, vital signs, and, of relevance for this study, surgical procedure logs. The EHR captures a median of 14 years of health data for patients within the MyCode® Community Health Initiative biorepository. Through the Geisinger-Regeneron DiscovEHR collaboration, whole exome sequence and genome wide genotype data are available from more than 92,000 MyCode® participants to date. These high dimensional clinical data linked to genetic data provide opportunities to conduct precision health research at an unprecedented scale that can lead to significant clinical insights. https://www.geisinger.org/precision-health/mycode

The details of MyCode Community Health Initiative have been described previously (Carey et al., 2016).

Genotyping was performed in two batches on the Illumina Infinium OmniExpress Exome array and GSA-24v1-0 array for Geisinger 60k and Geisinger 30k cohorts, respectively (Zhang et al., 2021). The Michigan Imputation Server was used to impute genotypes for both cohorts to HRC.r1-1 EUR reference genome (GRCh37 build) separately. Pre-imputation QC included sample call rate and marker call rate > 90%, HWE p value > 1e-15, MAF > 1%. A/T & G/C SNPs were removed if MAF > 0.4. SNPs with differing alleles, SNPs with > 0.2 allele frequency difference, SNPs not in HRC reference panel were also removed. Variants with imputation info score > 0.3 and MAC > 5 were included in the analyses. We used ICD-code based method to define OA cases and controls. We adopted a linear mixed model built in BOLT_LMM (N > 5000) or GEMMA (N < 5000) for the association tests while accounting for the relatedness and population structure (first 20PCs). PLINK1.9 was used for genetic data quality control and PC calculation.

##### Japan Study

The Japanese cohort of knee OA GWAS (disease cohort) consists of 900 cases and 3,400 controls. The cases were all symptomatic OA. They were diagnosed and recruited by expert orthopedic surgeons based on clinical and radiographic examination. All had clinical records for OA and radiographs (standing knee A-P). The controls were obtained from Biobank Japan. The genotyping was done by using Illumina HumanHap550v3 Genotyping BeadChip. After excluding cases with call rate of < 0.98, we applied SNP QC (call rate of ≥ 0.99 in both cases and controls and P value of Hardy-Weinberg equilibrium test of ≥ 1.0 × 10−6 in controls). Finally, 459,393 SNPs on autosomal chromosomes passed the QC filters (Nakajima et al., 2010).

##### deCODE

The deCODE genetics osteoarthritis study is an ongoing population based study in Iceland that was initiated in 1997. The study includes information on all subjects who have undergone total joint replacement in Iceland, and on osteoarthritis status from the Landspitali University Hospital electronic health records. Information on hand osteoarthritis patients is derived from a database of hand osteoarthritis patients that was initiated in 1972. Subjects have given blood or buccal samples to deCODE genetics biobank, which has gathered genotypic and medical data from more than 160,000 volunteer participants. https://www.decode.com/.

The details of OA definition and analyses have been described previously (Styrkarsdottir et al., 2014, 2018).

##### Rotterdam Study

The Rotterdam study is a large longitudinal population-based cohort study designed to study the risk factors for all major diseases of the elderly. The study started in 1991, has grown to up to 15000 individuals and has detailed phenotyping for cardiovascular, neurodegenerative, endocrine and locomoter diseases and more. For osteoarthritis, longitudinal X-rays on multiple joints and knee MRI’s are available (and scored), as well as information on joint pain. GWAS data are available for all individuals of the Rotterdam Study, as well as additional molecular layers (such as RNA, methylation, microbiome). Genomic studies in the Rotterdam Study are led by the Genetic Laboratory, Department of Internal Medicine of the ErasmusMC in Rotterdam (Ikram et al., 2020). http://www.epib.nl/research/ergo.htm, http://www.glimdna.org

Nurses’ Health Study and Nurses’ Health Study II: The Nurses’ Health Studies are among the largest prospective investigations into the risk factors for major chronic diseases in women (https://nurseshealthstudy.org/). The NHS is a prospective cohort study established in 1976. Blood samples were collected from a subset of participants in 1989-90. DNA was collected from cheek cells from another subset of participants in 2001-2004. The NHS II was established in 1989 to study a population younger than the original NHS cohort. Blood samples were collected on a subset of participants in 1996-1999. DNA from cheek cells was collected in 2006 from another subset of participants. Self-reported cases of total hip replacement from the NHS and NHS2 were analyzed for the GO meta-analysis.

##### TwinsUK

TwinsUK is the UK’s largest adult twin registry and the most clinically detailed in the world. Professor Tim Spector from King’s College London set up the cohort in 1992 to investigate the incidence of osteoporosis and other rheumatologic diseases in several hundred monozygotic (identical) twins. We now have almost 14000 identical and non-identical twins from across the UK, with ages between sixteen and one hundred and our research has expanded to include multiple diseases and conditions. TwinsUK aims to investigate the genetic and environmental basis of a range of complex diseases and conditions. Current research includes the genetics of metabolic syndrome, cardiovascular disease, the musculoskeletal system, aging, sight as well as how the microbiome affects human health. The TwinsUK cohort is now probably the most genotyped and phenotyped in the world. TwinsUK data have enabled multiple collaborations with research groups worldwide and the publication of research papers. https://twinsuk.ac.uk/

Details on OA definition as in Styrkarsdottir et al. (2014) and GWAS, QC imputation as described in Hysi et al. (2018) and den Hollander et al. (2017).

##### GARP and LLS studies

The GARP study is a prospective observational study in patients with familial generalized osteoarthritis, hand osteoarthritis and other osteoarthritis phenotypes. All patients (N = 380) have symptoms and definite radiological signs of osteoarthritis and represent an advanced disease state (Meulenbelt et al., 2008). https://www.lumc.nl/org/reumatologie/research/artrose/9031609354853/ The Leiden Longevity Study (LLS) consists of 420 Caucasian families with at least two long-lived siblings (men aged 89 years or above; women aged 91 years or above), the middle aged offspring and the partners of this offspring (Beekman et al., 2010). https://www.leidenlangleven.nl/en/home

##### Estonian Biobank

EGCUT has 52000 gene donors, who are all genotyped. In 2019, 100000 new donors will be collected and genotyped. All osteoarthritis cases were selected from Estonian Biobank which is a population-based biobank of the EGCUT. https://genomics.ut.ee/en

Osteoarthritis cases were chosen from ca 50000 participants of Estonian Biobank by using ICD 10 codes. To specify THR, TKR and TJR cases, the codes from NOMESCO Classification of Surgical Procedures were used in addition. The numbers for cases and controls used are provided in the attached table.

For genotyping, Illumina Human CoreExome, OmniExpress, 370CNV BeadChip and GSA arrays were used. Quality control included filtering on the basis of sample call rate (< 98%), heterozygosity (> mean ± 3SD), genotype and phenotype sex discordance, cryptic relatedness (IBD > 20%) and outliers from the European descent based on the MDS plot in comparison with HapMap reference samples. SNP quality filtering included call rate (< 99%), MAF (< 1%) and extreme deviation from Hardy–Weinberg equilibrium (p < 1 × 10−4). Imputation was performed using SHAPEIT2 for prephasing, the Estonian-specific reference panel (Mitt et al., 2017) and IMPUTE2 (Howie et al., 2009) with default parameters. Association testing was carried out with snptest-2.5.2, adjusting for 4 PCs, arrays, current age and sex (when relevant).

##### ALSPAC

Avon Longitudinal Study of Parents and Children (ALSPAC). Pregnant women resident in Avon, UK with expected dates of delivery 1st April 1991 to 31st December 1992 were invited to take part in the study. The initial number of pregnancies enrolled is 14,541 (for these at least one questionnaire has been returned or a “Children in Focus” clinic had been attended by 19/07/99). Of these initial pregnancies, there was a total of 14,676 fetuses, resulting in 14,062 live births and 13,988 children who were alive at 1 year of age. When the oldest children were approximately 7 years of age, an attempt was made to bolster the initial sample with eligible cases who had failed to join the study originally. As a result, when considering variables collected from the age of seven onward (and potentially abstracted from obstetric notes) there are data available for more than the 14,541 pregnancies mentioned above. The number of new pregnancies not in the initial sample (known as Phase I enrolment) that are currently represented on the built files and reflecting enrolment status at the age of 24 is 913 (456, 262 and 195 recruited during Phases II, III and IV respectively), resulting in an additional 913 children being enrolled. The phases of enrolment are described in more detail in the cohort profile paper and its update (see footnote 4 below). The total sample size for analyses using any data collected after the age of seven is therefore 15,454 pregnancies, resulting in 15,589 fetuses. Of these 14,901 were alive at 1 year of age. A 10% sample of the ALSPAC cohort, known as the Children in Focus (CiF) group, attended clinics at the University of Bristol at various time intervals between 4 to 61 months of age. The CiF group were chosen at random from the last 6 months of ALSPAC births (1432 families attended at least one clinic). Excluded were those mothers who had moved out of the area or were lost to follow-up, and those partaking in another study of infant development in Avon.

Ethical approval for the study was obtained from the ALSPAC Ethics and Law Committee and the Local Research Ethics Committees. Informed consent for the use of data collected via questionnaires and clinics was obtained from participants following the recommendations of the ALSPAC Ethics and Law Committee at the time.

#### Informed consent and study approval

##### arcOGEN

The arcOGEN study was ethically approved by appropriate review committees, and the prospective collections were approved by the National Research Ethics Service in the United Kingdom. All subjects in this study provided written, informed consent.

##### UKHLS

The UKHLS has been approved by the University of Essex Ethics Committee, and informed consent was obtained from every participant.

##### ARGO-Larissa

Verbal informed consent was given by all research participants prior to the collection of blood samples for the research. The research participant recruitment, consent process, and study protocol were approved by the Institutional Review Board of the University Hospital of Larissa and conform to the ethical principles set out in the Declaration of Helsinki (1975).

##### ARGO-Athens

The ARGO collection was conducted in three public hospitals (Attikon University General Hospital of Athens, Nea Ionia General Hospital Konstantopouleio, and KAT Hospital) and one private hospital (Lefkos Stavros General Hospital) in the city of Athens, Greece between February of 2015 and March 2017. All studies were approved by the relevant hospital Institutional Review Board and conducted in accordance with the principles set out in the Declaration of Helsinki. All patients provided written informed consent prior to participation.

##### UK Biobank

All participants signed consent to participate in UK Biobank and UK Biobank’s scientific protocol and operational procedures were reviewed and approved by the North West Research Ethics Committee (REC reference number 06/MRE08/65), North West Multicenter Research Ethics Committee (REC reference 11/NW/0382), the National Information Governance Board for Health and Social Care and the Community Health Index Advisory Group.

##### Hong Kong work -HKSpineOA

Informed consent was obtained from participants and ethics was approved by a local institutional board.

##### The Nord-Trøndelag Health Study (The HUNT Study)

All genotyped participants have signed a written informed consent regarding the use of data from questionnaires, biological samples and linkage to other registries for research purposes. The current study was approved by the Regional Committee for Medical and Health Research Ethics (REK) 2015/573.

##### Geisinger

MyCode Governing Board and an external Ethics Advisory Council approved the study and informed consent was obtained from all subjects as detailed in this reference: Carey et al. (2016).

##### Japan Study

The Ethical committee of RIKEN Yokohama Institute approved the study. Informed consent was obtained from all subjects.

##### deCODE

All participants who donated samples gave informed consent and the National Bioethics Committee of Iceland approved the study (VSN 14-148) which was conducted in agreement with conditions issued by the Data Protection Authority of Iceland. Personal identities of the participant’s data and biological samples were encrypted by a third-party system (Identity Protection System), approved and monitored by the Data Protection Authority.

##### Rotterdam Study

The Rotterdam Study has been approved by the Medical Ethics Committee of the Erasmus MC (registration number MEC 02.1015) and by the Dutch Ministry of Health, Welfare and Sport (Population Screening Act WBO, license number 1071272-159521-PG). The Rotterdam Study has been entered into the Netherlands National Trial Register (NTR; https://www.trialregister.nl) and into the WHO International Clinical Trials Registry Platform (ICTRP; https://www.who.int/ictrp/network/primary/en/) under shared catalog number NTR6831. All participants provided written informed consent to participate in the study and to have their information obtained from treating physicians. Nurses’ Health Study and Nurses’ Health Study II: This study was approved by the Institutional Review Boards of the Harvard T. H. Chan School of Public Health and Brigham and Women’s Hospital. Informed consent was obtained from all subjects for the collection of biospecimens for genotyping and the use of their genotype and de-identified data for research.

##### TwinsUK

Ethics approval was obtained from the Guy’s and St. Thomas’ Hospital Ethics Committee. Written informed consent was obtained from every participant. RAAK, GARP, LLS: Ethical approval for all studies was obtained from the medical ethics committee of the Leiden University Medical Center (RAAK: P08.239 and P19.013; GARP: P76.98; LLS P01.113) and informed consent was obtained from all participants.

##### ALSPAC

Ethical approval for the study was obtained from the ALSPAC Ethics and Law Committee and the Local Research Ethics Committees. Specific approval references for each clinic can be found at http://www.bristol.ac.uk/alspac/researchers/research-ethics/. Informed consent for the use of data collected at clinics was obtained from participants following the recommendations of the ALSPAC Ethics and Law Committee at the time. Consent for biological samples has been collected in accordance with the Human Tissue Act (2004).

##### Estonian Biobank

Our study has been reviewed and approved by the local ethics committee on Estonian Bioethics and Human Research, resolution nr 1.1-12/624. Informed consent was obtained from all subjects.

### Method details

#### Cohorts and phenotype definition

Genome-wide association analysis for osteoarthritis was performed across 21 cohorts (Table S1), for a total of 826,690 individuals (177,517 osteoarthritis patients). We defined 11 stratified osteoarthritis phenotypes: osteoarthritis at any site, osteoarthritis of the hip and/or knee, knee osteoarthritis, hip osteoarthritis, total joint replacement, total knee replacement, total hip replacement, hand osteoarthritis, finger osteoarthritis, thumb osteoarthritis and spine osteoarthritis (Figure 1; Table S1). Osteoarthritis was defined by either a) self-reported osteoarthritis, b) clinical diagnosed, c) ICD10 codes (Table S1) or d) radiographic as defined by the TREAT-OA consortium (Kerkhof et al., 2011), depending on the data available in the cohort (Table S1). Controls were OA-free or population-based with or without ICD code exclusions. An age exclusion if appropriate (preferable of 45 years and older) was applied at the discretion of each cohort. GWAS analysis were performed by each cohort, and adjusted for cohort specific covariates (Table S1).

#### Annotation of protein coding variants

For coding SNVs we considered only the following moderate to high impact annotations when weighting genes for prioritisation: transcript_ablation, splice_acceptor_variant, splice_donor_variant, stop_gained, frameshift_variant, stop_lost, start_lost, transcript_amplification, inframe_insertion, inframe_deletion, missense_variant, protein_altering_variant.

#### Mouse and human phenotypes

We investigated if any of the genes within 1Mb (upstream and downstream) of the 100 SNVs had a musculoskeletal or neuronal/pain phenotype in mouse knockouts using information from the Mouse Gene expression database (GDX) of the Mouse Genome Informatics (MGI) database (Bult et al., 2019; Finger et al., 2015). Mouse orthologs of the genes were extracted from Ensembl, using biomart (GRCh37, Version 69) (Yates et al., 2020). The MGI Batch Query was used to extract all mouse knockout phenotypes from the GDX for all of the investigated genes Using the MGI mouse phenotype ontology the following mouse knockout phenotypes were included for musculoskeletal phenotypes: skeleton phenotype, muscle phenotype and immune system phenotype. For mouse neuronal/pain phenotype the following MGI mouse phenotype ontology was included: nervous system phenotype. We also investigated if any genes had a musculoskeletal phenotype in mouse knockouts using information from https://www.hugedomains.com/domain_profile.cfm?d=boneandcartilage&e=com. Genes which had a mouse musculoskeletal or neuronal phenotype were reported (Table S10). We also investigated human skeletal genetic disorders. We used the Nosology and classification of genetic skeletal disorders (Mortier et al., 2019) to identify genes within 1Mb (upstream or downstream) of the 100 SNVs that had links to human musculoskeletal phenotypes. For human genes linked to monogenetic pain disorders, we downloaded the OMIM database (Amberger et al., 2015) (https://www.omim.org/) and extracted any genes containing phenotypes with the following key words: pain, pain and neuropathy, neuropathy. In addition, we also included genes linked to pain from the curated Human Pain Genetics Database (Meloto et al., 2018) (https://humanpaingenetics.org/hpgdb/) We then examined if any genes within 1Mb (upstream or downstream) of the 100 SNVs were included in those human pain gene lists. Human genes with a pain phenotype or link were reported (Table S10).

#### Additional phenotypes and endophenotypes

For additional information on the 100 identified osteoarthritis associated SNVs, we examined their association in several osteoarthritis endophenotype and structural phenotype GWAS studies. The osteoarthritis definition and related structural phenotypes were defined based on radiographs in the Rotterdam Study cohorts of the hip, hand, finger, thumb and knee joints (n = 5,634 to 9,276). We have used the following radiographic measurements to create (semi)-quantitative endophenotypes for the hip, knee, hand, finger and thumb joints: Joint Space Narrowing (JSN) (0–3 scoring), Joint Space Width (JSW) (mm), Osteophytes (0–3 scoring), and Kellgren-Lawrence (KL)-score (0–5). Using these measurements we have defined the following structural OA phenotypes: Finger/Hand/Thumb/Knee/Hip JSN sum score, osteophyte sum score, KL sum score and Hip JSW.JSW was assessed at pelvic radiographs in anterior-posterior position and measured in mm, along a radius from the center of the femoral head. Within the Rotterdam Study, a 0.5 mm graduated magnifying glass laid directly over the radiograph was used to measure the joint space width of the hip joints. Acetabular dysplasia was measured using the Center-Edge angle or also known as the Wiberg (CE-angle). The angle was measured using statistical shape model (SSM) software. A continuous phenotype was used for the CE-angle, because of the normal distribution of the measured angles. Since the CE-angle of the right hip and the left hip has a high correlation (Pearson correlation coefficient 0.68), only the CE-angle of the right hip was used in our GWAS. Minimal Joint Space Width (mJSW) GWAS data was taken from Castaño-Betancourt et al. (2016). Summary statistics for Bone Size as measured by DXA were taken from Styrkarsdottir et al. (2019).

#### Cartilage-type specific effect

To investigate if any of the high-confidence effector genes show a different expression in osteophytes, indicating a potential role in repair mechanisms in response to joint cartilage degeneration, we investigated if they showed significant (0.1% FDR) differential gene expression, methylation or differential protein abundance in osteophytic cartilage compared to low-grade (intact) cartilage in a within-individual matched analysis from 9 individuals who had undergone THR for primary osteoarthritis (Steinberg et al., 2018) (Table S12).

#### Effect on intervertebral disc degeneration

To identify if any of the high confidence effector genes code for proteins that are implicated in disc degeneration, we investigated if the proteins were differentially abundant in recently published spatiotemporal proteomics atlas of human intervertebral discs (Tam et al., 2020). Eight effector genes demonstrate differential protein abundance in a comparison between intervertebral discs from a younger (16 year old male) and an older (59 year old male) individual with no reported scoliosis or degeneration (Table S12).

#### Monogenic and rare human diseases

We scanned the Human Phenotype Ontology (HPO) database (Köhler et al., 2021), which is currently being developed using the medical literature, DECIPHER (https://www.deciphergenomics.org/about/overview) (Firth et al., 2009), OMIM (https://omim.org/) (Amberger et al., 2015) and Orphanet (https://www.orpha.net/consor/cgi-bin/index.php?lng=EN), to examine if any of the high confidence effector genes are implicated in phenotypic abnormalities of monogenic and rare human diseases. Fifty-one genes are involved in diseases related to skeletal development, joint degeneration, adipogenesis, muscle function, neuronal function, immune response and inflammation (Figure 3; Table S12).

### Quantification and statistical analysis

#### Meta-analysis

GWAS summary statistics from all cohorts were collected and checked to contain all the data needed for the meta-analysis. The quality control (QC) was performed centrally by using EasyQC (Winkler et al., 2014). Briefly, missing data, mono-allelic SNVs, nonsensical values (p > 1, infinite beta’s etc.) and duplicates were removed from the data. We excluded variants with poor imputation quality (R^2^ < 0.3), if the effective sample size was < 20 and if the minor allele count was < 6. Allele coding was harmonized across cohorts (A/T/C/G or I/D). Allele frequency was checked against the imputation reference (HRC http://www.haplotype-reference-consortium.org/) (McCarthy et al., 2016) or 1000G http://www.internationalgenome.org/) to identify possible allele coding errors. P values were checked to match the corresponding beta values. Cleaned data was used as input for the meta-analysis. Meta-analysis was performed using inverse variance weighting in METAL (Willer et al., 2010). Genomic control was performed on all datasets, except those which had already carried out genomic-control adjustments prior to centralized QC and meta-analysis. Genome-wide significance threshold was set at p < 1.3x10^−8^, corrected for multiple testing (see significance threshold section below). For each phenotype we only considered variants reported in at least 2 cohorts with the same direction of effect with a minimum MAF > = 0.0001 in any contributing cohort. We repeated the same procedure to perform two sensitivity analyses: a) we excluded from the meta-analyses the largest contributing dataset, UK Biobank (https://www.ukbiobank.ac.uk/) (Bycroft et al., 2018) and b) East Asian ancestry-only meta-analyses for the 4 osteoarthritis phenotypes (spine, knee, knee and/or hip, and osteoarthritis at any site) that included East Asian cohorts. To summarize the significance of the signals that have supportive evidence in East Asian ancestry-only meta-analysis, we conducted a binomial test (N = 10 SNVs with concordant direction and p ≤ 0.05, N = 77 SNVs tested, and 0.025 is the expected proportion of SNVs at p ≤ 0.05 and with the same direction of effect). As a sensitivity analysis, we excluded the largest contributing dataset in which the majority of previously-reported loci originate (UK Biobank; up to 68,621 osteoarthritis cases and 247,846 controls) from the meta-analyses.

#### Sex-differentiated meta-analysis

The meta-analyses and QC steps described above were repeated for males and females separately in a subset of cohorts. We then combined the resulting association summary statistics (male-specific meta-analysis, consisting of up to 56,462 cases and 153,808 controls, and female-specific meta-analysis, consisting of up to 90,838 cases and 192,697 controls) to conduct a sex-differentiated test of association and a test of heterogeneity in allelic effects, as implemented in GWAMA (Mägi et al., 2010; Mägi and Morris, 2010). This method allows for heterogeneity of allelic effects in magnitude and/or direction between males and females and offers substantial gains in power to detect SNV associations. The genome-wide significance threshold was set at p < 1.3x10^−8^, corrected for multiple testing. Heterogeneity in allelic effect sizes was assessed with Cochran’s Q statistic and the significance threshold was set at p < 0.016, corrected for the 3 independent new signals identified across the 11 osteoarthritis phenotypes (Table S5).

#### Early-onset osteoarthritis meta-analysis

We carried out a meta-analysis of early osteoarthritis, defined as age at onset younger than 45 years of age, across 3 cohorts (Estonian Biobank, HUNT & UK-Biobank) with age at onset information available. The analysis was conducted on 6,838 early-onset osteoarthritis patients and 41,449 controls in only one of the 11 phenotype definitions used in the main meta-analysis (osteoarthritis at any site, abbreviated as EarlyAllOA). The QC and meta-analysis steps of the main meta-analysis were repeated for early-onset meta-analysis and the genome-wide significance threshold was set at p < 5x10^−8^.

#### Significance threshold

The testing of *M* = 11 osteoarthritis phenotypes in this study needed to be taken into account in the interpretation of genome-wide statistical significance. Applying a Bonferroni correction would be inherently conservative as this method assumes independence among the tests considered. Therefore, we first used LD Score regression method (Bulik-Sullivan et al., 2015a, 2015b) (https://github.com/bulik/ldsc/) with genome-wide meta-analysis summary statistics to estimate the genetic correlation matrix between the 11 osteoarthritis traits (Table S6) and then calculated the effective number of independent traits (M_eff) from the eigenvalues λ_i of the correlation matrix (Li et al., 2012):

Meff=M−∑i=1M[I(λi>1)(λi−1)]

For the M = 11 osteoarthritis phenotypes in this study, M_eff = 4.6565. The threshold corrected for the effective number of traits to report genome-wide significance is p < 1.3x10^−8^.

#### Statistical independence

To define independent signals for each osteoarthritis phenotype (Table S3), we used the clumping function in PLINK 1.9 (Purcell et al., 2007) with the following parameters: (a) significance threshold for index variants: p < 1.3x10^−08^, (b) LD threshold for clumping: 0.10, and (c) physical distance threshold for clumping: 1Mb (2Mb window around the index variant). LD calculations were based on the full UK Biobank imputed set. To test that the index variants defined by clumping were statistically independent, we performed an approximate stepwise model-selection procedure, as implemented by COJO in GCTA (Yang et al., 2011, 2012) (https://cnsgenomics.com/software/gcta/). A signal in a region was defined as independent if its P value of association in the stepwise regression was less than the adjusted genome-wide significant threshold (p < 1.3x10^−8^).

To define independent signals across the 11 osteoarthritis phenotypes (Tables 2 and S3), we performed reciprocal approximate conditional analyses, as implemented by COJO in GCTA (Yang et al., 2012; Yang et al., 2011), of each independent variant of one osteoarthritis phenotype conditioned on each independent variant of the other osteoarthritis phenotypes within 1-Mb region. Two signals were considered dependent if the P value for either signal conditioned on the other was either ≥ 1x10^−7^, or attenuated by at least 2 orders of magnitude. Among dependent variants, the one with the lowest P value was classified as independent. Using an approximate conditional and joint multiple-SNP analysis, as implemented by COJO in GCTA (Yang et al., 2012; Yang et al., 2011), we investigated the statistical independence between index signals per osteoarthritis phenotype and previously reported osteoarthritis variants within a 1-Mb region. In Tables 1, 2, and S2 the index variant was classified as a new association if it had a conditional p ≤ 1x10^−7^ or the P value difference between conditional and unconditional analysis increased by more than two orders of magnitude. Index variants were classified as known if they have previously been reported or the association signal disappeared after conditioning on the variant of a previously reported locus.

#### Polygenic-risk-score analyses

PRS were created for all osteoarthritis phenotypes with 3 different approaches by using 2 different software, PRSice2 (Choi et al., 2020; Choi and O’Reilly, 2019) which has a P value thresholding shrinkage strategy and LDpred (Vilhjálmsson et al., 2015) that uses a Bayesian approach to polygenic risk scoring. First, we recreated summary statistics from the main sex-combined meta-analyses excluding arcOGEN samples (Table S1), in order to use the largest possible discovery sample for calculating the weights. PRS were created for the arcOGEN cohort individuals for all available phenotypes (Table S4) using raw genotype data in the software PRSice2 with the binary trait settings. The P value thresholds ranging from 1.3x10^−8^ to 1.0x10^−4^ with LD clumping parameters of R^2^ = 0.1 over 1Mb windows and 10,000 permutations to account for the overfitting. As arcOGEN doesn’t have data for spine osteoarthritis, hand osteoarthritis, thumb osteoarthritis and finger osteoarthritis, we calculated PRS in the UK Biobank samples. For the weights we used the main sex combined meta-analysis excluding UK Biobank individuals. We performed the analysis in PRSice2 using P value thresholds from 1.3x10^−8^ to 1.0x10^−4^ with LD clumping parameters of R^2^ = 0.1 over 1Mb windows and the binary trait setting. In our third approach, deCODE was used as the target dataset since all 11 phenotypes are available and the loss of power in the base dataset wouldn’t be as great as if we had excluded the UKBB dataset. We used PRS analyses for one osteoarthritis trait to investigate its predictive power for another osteoarthritis trait (Table S4). We used effect estimates based on meta-analysis for osteoarthritis excluding deCode. The risk scores were calculated using genotypes for about 600,000 well-imputed autosomal markers. We estimated LD between markers using 4,000 phased Icelandic samples and used this LD information to calculate adjusted effect estimates using LDpred. We created several PRS assuming different fractions of causal markers (the P parameter in LDpred), and selected the PRSs that best predicted the trait itself. The correlation between the PRS and traits was calculated using logistic regression in R (v3.5) (R Core Team, 2019) adjusting for principal components, sex and year of birth by including them as covariates in the analysis. Variance explained is estimated using Nagelkerke R^2^. We binned the UK Biobank individuals based on their PRS into deciles and we calculated the odds ratio (OR), 95% CI and P value (Fisher’s Exact test) for individuals in the top decile compared to the bottom decile (Table S4).

We generated PRS in the genome-wide meta-analysis excluding the deCODE dataset and used univariate linear regression to test the predictiveness of the scores on age at joint replacement in the deCODE cohort (Table S4).

To investigate PRS predictability of patient strata, we assigned the high confidence effector genes into 6 broad areas of osteoarthritis biological action; skeletal development, joint degeneration, immune function and inflammation, neuronal function and development, muscle function and adipogenesis (Figure 3A; Table S13). For each group the lead SNV for each effector gene member was used to construct a PRS. A meta-analysis without UK Biobank included was used as the base data and UK Biobank was used as the target using PRSice2 (Choi et al., 2020; Choi and O’Reilly, 2019) (Table S4).

We investigated associations between osteoarthritis PRS and bone mineral density (BMD) and body mass index (BMI) trajectories in Avon Longitudinal Study of Parents and Children (ALSPAC) study (Boyd et al., 2013; Fraser et al., 2013), by analyzing 24,844 BMI, total body less head BMD (TBLH BMD), total body fat mass (TBFM) and total body lean mass (TBLM) observations from 6,263 individuals.

##### Outcome assessment

BMI, total body less head BMD (TBLH BMD), total body fat mass (TBFM) and total body lean mass (TBLM) were measured at the following clinics for adolescents: a) Age 9 (mean age 9.9), b) Age 11 (mean age 11.8), c) Teen focus 2 (mean age 13.8), d) Teen focus 3 (mean age 15.5), e) Teen focus 4 (mean age 17.8) and f) Focus at 24 (mean age 24.5). TBFM and TBLM indices were generated by dividing TBFM or TBLM by height^2^. Mean BMI, BMD, TBFM and TBLM at each clinic are presented in Table S4. SNVs associated with osteoarthritis at genome-wide significance were included in the scores. Unweighted polygenic risk scores were generated by summing the dosage of the osteoarthritis risk-increasing alleles. Weighted polygenic risk scores were generated by multiplying the dosage of the risk-increasing alleles by the log odds and summing across all alleles. PRS were generated for the SNPs associated with osteoarthritis at any site, hip osteoarthritis and knee osteoarthritis.

Associations between PRS and BMI trajectories were determined using mixed-effects linear spline regression modeling. 5 knot points were generated at the mean age at each clinic starting at mean age 11.8. PRS-by-spline interaction terms were included to determine if PRS affects the rate of BMI change between time points. Models were adjusted for sex and 4 principal components (PCs). Age (centered at 9.9, the mean age at the first clinic) and individual ID were included as random effects. Analyses were repeated stratified by gender.

As a sensitivity analysis, analyses were repeated with DXA scans coded as having artifacts excluded, as well as DXA scans coded as having major positioning errors.

##### Correction for multiple testing

We analyzed six different exposures (weighted and unweighted PRS for osteoarthritis at any site, hip osteoarthritis and knee osteoarthritis) and five different outcomes (BMI, TBLH BMD, TBFMi, TBLMi and height). However, the weighted and unweighted PRS are highly correlated and BMD/TBFMi/TBLMi and height are all components of BMI. Therefore, our corrected P value threshold was determined as 0.05/3 = 0.017. Removing observations extracted from DXA scan images with artifacts or positioning errors did not affect conclusions. Diagnostic tests did provide some evidence of heteroskedasticity and non-normality of residuals for BMI and TBFMi, but log-transforming these outcomes did not alter results or improve residuals. The study website contains details of all the data that is available through a fully searchable data dictionary and variable search tool (http://www.bristol.ac.uk/alspac/researchers/our-data/).

#### Genetic signals across phenotypes

Results from all independent lead SNVs (n = 100) across all osteoarthritis phenotypes were extracted from the full meta-analysis results. All OR were calculated on the minor allele (allele frequency < 50%), and SNVs with a MAF < 1% were excluded (n = 6). For all the remaining SNVs (n = 94) the OR for each osteoarthritis phenotype GWAS was plotted in a heatmap, together with the corresponding association *P* (Figure 2). All figures were plotted using R and adjusted for publication quality using Adobe illustrator.

We also created three classification groups: 0 = Weight bearing joints only (hip and/or knee, knee, hip, total joint replacement, total knee replacement, total hip replacement and spine), 1 = Both, 2 = Non-weight bearing joints only (hand, finger, thumb). Osteoarthritis at any site wasn’t included in this analysis as it wasn’t clear which osteoarthritis subphenotype was leading the signal. In Table 2 and Figure 2 each of the 100 independent genome wide significant SNVs was assigned to the above groups only if it wasn’t nominally significant (p > 0.05) for any of the other phenotypes in the other classification groups, resulting in 86 SNVs to be further analyzed.

#### Genetic correlation

We estimated the genetic correlation between osteoarthritis traits and secondary traits using the cross-trait LD Score regression method as implemented in LDHub (Bulik-Sullivan et al., 2015a, 2015b) (https://github.com/bulik/ldsc). We used results for about 1.1 million well imputed variants, and for LD information we used pre-computed LD scores for European populations (https://alkesgroup.broadinstitute.org/LDSCORE/eur_w_ld_chr.tar.bz2). LD scores for the East Asian populations could not be calculated as the LD Score method requires a sample size of > 4000 individuals.

To avoid bias due to overlapping samples, we calculated the genetic correlation between equally sized, non-overlapping subgroups of the sample sets from the meta-analysis of Icelandic, Norwegian and USA samples and UK, Dutch, Estonian and Greek samples (Table S6). The results of the two analyses were subsequently meta-analyzed. For genetic correlations with other traits, we calculated the genetic correlation between a meta-analysis of UK, Dutch, Estonian and Greek samples and the Icelandic GWAS summary statistics for each secondary trait, and also between a meta-analysis of Icelandic, Norwegian and USA samples and UKBB GWAS summary statistics for each secondary trait. The results of the two analyses were subsequently meta-analyzed. We also analyzed the genetic correlation between the osteoarthritis subtypes, we split the sample-sets of the meta-analysis in two equally sized groups and performed LD score regression between the two groups for each subtype (Table S6).

#### Fine-mapping

For each independent signal we included all variants within 1Mb of the index variant. In situations where there was more than 1 osteoarthritis signal in the region we used the conditional summary statistics of the meta-analysis conditioned on all other signals. We calculated Wakefield’s asymptotic Bayes factors (Wakefield, 2009) and we determined the posterior probability of each variant being causal. To produce a 95% credible set of variants we ranked according to posterior probability and included those variants with the highest probability of being causal until the shared probability was at least 95%. Some regions were large therefore we considered only variants in the 95% credible with a posterior probability of causality > 3% (Table S8).

#### eQTL colocalization

For cis-eQTL colocalization we used summary statistics of SNPs from 48 human tissues from the GTEx v7 (Battle et al., 2017) (https://www.gtexportal.org/home/). For each signal and each tissue we included genes that contained at least 1 eQTL (using a threshold of < 5% false discovery rate) in GTEx and that overlapped 100kb either side of our signal. For the colocalization analysis we included all variants in common between the meta-analysis conducted here and the GTEx cis-eQTL analysis with the exception of indels. We used the Bayesian statistical methodology (https://github.com/tobyjohnson/gtx/blob/526120435bb3e29c39fc71604eee03a371ec3753/R/coloc.R) which implements the method of Giambartolomei et al. (2014). This method evaluates whether the GWAS and molecular QTL associations best fit a model in which the associations are due to a single shared variant, summarized by the posterior probability (PP). Evidence for colocalization was assessed using the PP4 indicating that there is an association for both traits and they are driven by the same causal variant. A PP4 > 0.8 was considered evidence for colocalization (Table S8).

#### Tissue specificity

Most complex disease risk variants are thought to exert their risk by affecting regulation of expression of a target gene in a tissue and cell-specific context (Maurano et al., 2012; Kundaje et al., 2015). Osteoarthritis affects multiple tissues within the joint, most notably the cartilage and bone, but there is also evidence for involvement of the synovium, and possibly the muscles and tendons of the joint (Brandt et al., 2006). To identify possible further osteoarthritis target tissues, we selected all independent genome-wide significant SNVs across osteoarthritis GWAS (n = 100). For each signal we investigated the lead SNV and all fine-mapped SNVs (95% posterior probability) (n = 542) to see if they colocalized with active enhancer histone marks as defined by the ROADMAP epigenomics project (Kundaje et al., 2015) (http://www.roadmapepigenomics.org/). All tissue and cell types available in the ROADMAP epigenomics and ENCODE project were used (n = 127) (Snyder et al., 2020; Kundaje et al., 2015). For each lead SNV and their fine-mapped SNVs, the percentage of SNVs located in active enhancer marks was calculated. For the enrichment analysis a background value was calculated (1000 permutations) using 100 random SNVs selected from 1000 Genomes Project (Auton et al., 2015) (MAF > 0.05). For these 100 random SNVs and all SNV in high LD (R^2^ = 0.8, LD based on 1000 Genomes Project) their percentage of colocalization with enhancer histone marks was calculated. Once all background permutations were done, an average of all results was taken as the final background values. Enrichment was calculated for each osteoarthritis phenotype and investigated cell type separately, by using the two-proportions Z-test. The significance for enrichment was set to genome-wide significance (p < 1.3x10^−8^) (Figure S1). As the analysis is highly dependent on the number of variants included (power), significance was only based on enrichment analysis including all 100 independent SNVs across osteoarthritis phenotypes. For the eQTL colocalization using GTEx tissues, we considered the following GTEx tissues as possible osteoarthritis target tissues, based on the tissue specificity analysis: Adipose, Brain, Heart, Lung, Muscle, Nerve, Ovary, Placenta, Skin, Stomach, Cultured fibroblasts, Adrenal gland, and Breast tissue. In the section ‘Amassing evidence to identify effector genes’, genes that had an eQTL colocalization in one of these tissues received an additional scoring point in the lines of evidence (Tables 3 and S10).

#### Causal inference analysis

Two-sample Mendelian randomization (MR) was applied to understand the association between plasma proteins on osteoarthritis. In the MR analysis, 1640 proteins in up to 6,000 individuals were treated as the exposure and the 11 osteoarthritis phenotypes as the outcomes. The genetic instruments of the plasma proteins were obtained from Zheng et al. (2019), where the conditional independent pQTLs were pooled from 5 recent GWAS of plasma proteins (Emilsson et al., 2018; Folkersen et al., 2017; Suhre et al., 2017; Sun et al., 2018; Yao et al., 2018). The genetic instruments were further split into two groups: 1) *cis*-acting pQTLs within a 500Kb window from each side of the leading pQTL of the protein were used for the MR analysis; 2) *trans*-acting pQTLs outside the 500kb window of the leading pQTL were designated as trans instruments. For the MR analysis, the meta-analysis summary statistics of osteoarthritis including UK Biobank participants were used as outcomes. We selected a P value threshold of 0.05, corrected for 11 osteoarthritis phenotypes and the number of independent tests, as our threshold for prioritising MR results for follow up colocalization analyses (number of tests = 18.030; p < 2.77x10^−6^) (Table S7).

For 28 protein-osteoarthritis associations that survived the multiple testing threshold in the MR analysis, we further conducted colocalization (Giambartolomei et al., 2014) analysis to distinguish causal effects from genomic confounding due to linkage disequilibrium. A colocalization probability more than 80% in this analysis would suggest that the two association signals are likely to colocalize within the test region. Colocalization analysis was applied to both *cis* and trans pQTLs. For protein and phenotype GWAS lacking sufficient SNP coverage or missing key information (e.g., allele frequency or effect size) in the test region, we conducted a LD check (Zheng et al., 2019) for the sentinel variant for each pQTL against the 30 strongest SNPs in the region associated with the phenotype as an approximate colocalization analysis. R^2^ of 0.8 between the sentinel pQTL variant and any of the 30 strongest SNPs associated with the phenotype was used as evidence for approximate colocalization (Table S7).

Nine protein-osteoarthritis associations showed reliable MR and colocalization evidence (Bonferroni corrected, p < 2.77x10^−6^ and colocalization probability > 70%) for a total of six proteins on seven osteoarthritis phenotypes (Table S7). Of the eight protein quantitative trait loci (pQTLs) used as genetic predictors of these six proteins, five were in strong LD with missense variants (R^2^ > 0.8). As missense variants may cause epitope-binding artifacts, we also evaluated the effect of these eight pQTLs on other molecular traits: DNA methylation (meQTL) (Gaunt et al., 2016) and gene expression (whole blood eQTLs from eQTLGen and all tissues eQTLs from GTEx) (Aguet et al., 2019; Võsa et al., 2018). Six of the eight pQTLs were also *cis* meQTLs and *cis* eQTLs in the same region, in which four pQTLs are in LD (R^2^ > 0.3) with the top meQTL and eQTL in the region (Table S7).

#### RNA sequencing analysis of the RAAK cohort

We performed an investigation for all genes within 1Mb (upstream and downstream) of the 100 SNVs in preserved and lesioned cartilage and subchondral bone samples from the same donor were obtained from the Research in Articular osteoArthritis Cartilage (RAAK) study consisting of patients with osteoarthritis who underwent joint replacement surgery due to an end-stage disease (Coutinho de Almeida et al., 2019; den Hollander et al., 2019; Ramos et al., 2014). Total RNA from articular cartilage and subchondral bone was isolated using QIAGEN RNeasy Mini Kit (QIAGEN, GmbH, Hilden, Germany). Paired-end 2 × 100 bp RNA-sequencing (Illumina TruSeq RNA Library Prep Kit, Illumina HiSeq2000 and Illumina HiSeq4000) was performed. Strand specific RNA-Seq libraries were generated which yielded a mean of 20 million reads per sample. More details on mapping and quality control (QC) from cartilage are previously described (Coutinho de Almeida et al., 2019; den Hollander et al., 2019). Methods of subchondral bones RNA sequencing have been previously described (Tuerlings et al., 2020). After QC, 35 paired cartilage samples (N = 70) and 24 paired subchondral bone samples (18 paired knee and 6 paired hip samples) remained for further differential expression analysis. Normalization and statistical framework was performed using the DESeq2 v1.20 R package. A general linear model (GLM) assuming a negative binomial distribution was applied followed by a paired Wald-test between preserved and lesioned osteoarthritis cartilage and subchondral bone samples. Benjamini-Hochberg multiple testing corrected P values with significance cut-off of 0.05 are reported as False Discovery Rate (FDR).

#### RNA sequencing and proteomic analysis of the UK cohort

We performed an *in-silico* investigation for all genes within 1Mb (upstream and downstream) of the 100 SNVs in 38 individuals for which differential expression and differential abundance was available, as described previously (Steinberg et al., 2017; Tachmazidou et al., 2019). Briefly: DNA, RNA, and protein was extracted from matched intact and degraded cartilage samples from 38 patients undergoing total joint replacement surgery: 29 knee and 9 hip osteoarthritis patients. From each patient, 2 paired cartilage samples were taken; a sample with a low Osteoarthritis Research Society International (OARSI) grade which signifies healthy or macroscopically intact cartilage tissue (intact) and a sample with a high OARSI grade, which denotes highly degenerated (degraded) cartilage tissue. All patients provided full written informed consent before participation. The human biological samples were sourced ethically and their research use was in accord with the terms of the informed consents under an Institutional Review Board (IRB)- or Ethics Committee (EC)-approved protocol. Proteomics analysis was performed on intact and degraded cartilage samples from 24 individuals and gene expression analysis on samples from all 38 patients. For the proteomics we performed LC-MS analysis on the Dionex Ultimate 3000 UHPLC system coupled with the Orbitrap Fusion Tribrid Mass Spectrometer. Abundance values were normalized by the sum of all protein abundances in a given sample, then log2-transformed and quantile normalized. We restricted the analysis to 3917 proteins that were quantified in all samples. Differential abundance was performed using a within-individual paired sample design in limma in R (Ritchie et al., 2015). RNA sequencing was performed on the Illumina HiSeq 2000 (75bp paired-end read length). We determined the gene-level counts from transcript-level quantification using salmon 0.8.219 with GRCh38 cDNA assembly release 87 downloaded from Ensembl. Limma-voom (Law et al., 2014) was used to remove heteroscedasticity from the estimated expression data. We tested genes for differential expression using limma in R (with lmFit and eBayes), based on a within-individual paired sample design. For the proteomics and RNA sequencing significance was defined at 5% Benjamini-Hochberg FDR to correct for multiple testing.

For the cartilage RNaseq differential analysis investigation we combined data from the two different sources (detailed above): 35 paired samples from the RAAK study (Coutinho de Almeida et al., 2019; den Hollander et al., 2019; Ramos et al., 2014) and 38 paired samples from a UK cohort (Steinberg et al., 2017; Tachmazidou et al., 2019). We only considered genes that were significantly (FDR < 0.05) differentially expressed in both studies (RAAK cohort and UK cohort) with the same direction of effect (Table S9).

#### Subchondral bone differential gene expression

We performed an *in-silico* investigation for all genes within 1Mb (upstream and downstream) of the 100 SNVs for gene expression in subchondral bone compared to intact cartilage. Briefly, knee joint samples were collected in 11 Han Chinese patients from the Taiwan OA cohort that had undergone total knee replacement surgery (TKR). The subchondral bone tissues underneath the intact and eroded cartilage were obtained as previously described (Chou et al., 2013b). RNA was extracted as described (Chou et al., 2013a). RNA (400ng) per sample were used for cRNA synthesis and amplification. Cyanine 3-labeled cRNA was then purified and hybridized to Agilent whole human genome 44 k microarray chips (Agilent Technologies) according to the manufacturer’s instructions (Agilent Technologies, Santa Clara, CA). The array signal intensities were analyzed by the Agilent GeneSpring GX software (version 11.5; Agilent Technologies). Gene expression values were normalized using quantile normalization; probes with low signal intensities were excluded by setting the filter above 32. The normalized values were log transformed and compared using the t test. Differentially expressed genes were defined at ≥ 2 fold-change with Benjamini-Hochberg corrected p ≤ 0.05 (Table S9).

For the subchondral bone RNaseq differential analysis investigation we combined data from the two different sources (detailed above): 24 paired samples from the RAAK study (Coutinho de Almeida et al., 2019; den Hollander et al., 2019; Ramos et al., 2014) and 11 Han Chinese patients from the Taiwan OA cohort. We only considered genes that were significantly (FDR < 0.05) differentially expressed in both studies (RAAK cohort and UK cohort) with the same direction of effect (Table S9).

#### Phenome-wide analysis

To cross-reference all independent lead SNVs across all osteoarthritis phenotypes with many other traits and diseases. We queried the PhenoScannerV2 (Kamat et al., 2019) (http://www.phenoscanner.medschl.cam.ac.uk/) database through the web-based tool on 15/02/2021 and at the time it contained > 5,000 genotype-phenotype association datasets from large-scale genetic association studies. Only associations with P value less than the genome wide significance threshold (p < 5x10^−8^) were included in the analysis and proxies were not requested.

#### Prioritized genes in the Druggable Genome

We examined the druggability status for the 637 prioritized genes (Table S10), using the druggable gene set as defined by Finan et al. (2017). The approach to define the Druggable Genome extended the concept of Mendelian randomization studies for drug development from individual targets to the whole genome. This druggable genome contained 4,479 genes and it was divided in three tiers of druggable gene sets corresponding to position in the drug development pipeline. Tier 1 (1427 genes) included efficacy targets of approved small molecules and biotherapeutic drugs as well as clinical-phase drug candidates. This tier incorporated the targets of approved drugs (licensed drugs) and drugs in clinical development. Proteins that are targets of approved small-molecule and biotherapeutic drugs were identified using manually curated efficacy target information from release 17 of the ChEMBL database (Gaulton et al., 2017). Tier 2 was composed of 682 genes encoding targets with known bioactive drug-like small-molecule binding partners as well as those with ≥ 50% identity (over ≥ 75% of the sequence) with approved drug targets. This tier incorporated proteins closely related to drug targets or with associated drug-like compounds. Proteins closely related to targets of approved drugs were identified through a BLAST search (blastp) of Ensembl peptide sequences against the set of approved drug efficacy targets identified from ChEMBL (Gaulton et al., 2017) previously. Tier 3 contained 2370 genes encoding secreted or extracellular proteins, proteins with more distant similarity to approved drug targets, and members of key druggable gene families not already included in tier 1 or 2 [G protein (heterotrimeric guanine nucleotide–binding protein)–coupled receptors (GPCRs), nuclear hormone receptors, ion channels, kinases, and phosphodiesterases].This tier was further subdivided to prioritize those genes that were in proximity (±50 kbp) to a GWAS SNP from GWAS catalog and had an extracellular location (Tier 3A). The remainder of the genes was assigned to Tier 3B. To test if there is enrichment of genes with supporting evidence in the druggable genome database, we conducted a binomial test (N = 205 prioritized genes in the database, N = 637 prioritized genes in total, N = 4479 genes included in the database out of 20330 coding genes tested).

To glean further insight into the detailed structured information about drugs and drug targets of the identified effector genes in tier 1 or 2 of the druggable genome database, we integrated information from the DrugBank online database (https://www.drugbank.com) (Wishart et al., 2018), accessed between 3–10 March 2021 (version 5.1.8, released 2021-01-03). Of the 71 tier 1 genes, 58 (23 newly associated with osteoarthritis) have info in DrugBank, of which 18 are high-confidence genes (7 newly associated with osteoarthritis).

#### Pathway analyses

We assessed pathway and gene set signal enrichment for the 637 genes with at least one line of evidence and, separately, for the 77 high-confidence putative effector genes (Table S13). Pathway and gene set enrichment analysis were performed using the ConsensusPathDB-human enrichment software (http://cpdb.molgen.mpg.de/) (Kamburov et al., 2011). We used the Wikipathways enrichment and the Gene Ontology Biological processes gene set. Enriched gene sets required a minimum of 5 genes to overlap with the examined gene set. The significance threshold was set at FDR < 0.05.

We also performed 3 additional stratified pathway analysis on (I) all SNVs associated with only weight bearing joints (165 genes), (II) all SNVs associated with only non-weight bearing joints (24 genes), and (III) all SNVs associated with both weight bearing and non-weight bearing joints (155 genes) by using the Gene2Func function of FUMA (Functional Mapping and Annotation of Genome-Wide Association Studies, https://fuma.ctglab.nl/) (Watanabe et al., 2017). Analysis was performed as described in Watanabe et al. (2017) using the following settings: all known genes and transcripts were included in the background gene set, we included the MHC-region, a minimum of 5 genes needed to overlap with the examined gene sets and the significance threshold was set at FDR < 0.05 (Table S13).

## Consortia

The members of arcOGEN Consortium are John Loughlin, Nigel Arden, Fraser Birrell, Andrew Carr, Panos Deloukas, Michael Doherty, Andrew W. McCaskie, William E.R. Ollier, Ashok Rai, Stuart H. Ralston, Tim D. Spector, Ana M. Valdes, Gillian A. Wallis, J. Mark Wilkinson, and Eleftheria Zeggini.

The members of the HUNT All-In Pain Consortium are Amy E. Martinsen, Cristen Willer, Egil Andreas Fors, Ingunn Mundal, Knut Hagen, Kristian Bernhard Nilsen, Marie Udnesseter Lie, Sigrid Børte, Ben Brumpton, Jonas Bille Nielsen, Lars G. Fritsche, Wei Zhou, Ingrid Heuch, and Kjersti Storheim.

The members of the ARGO-Athens Consortium are Eleni Zengini, George Alexiadis, Evangelos Tyrpenou, Athanasios Koukakis, Dimitrios Chytas, Dimitrios Stergios Evangelopoulos, Chronopoulos Efstathios, Spiros Pneumaticos, Vasileios S. Nikolaou, J. Mark Wilkinson, George C. Babis, Konstantinos Hatzikotoulas, and Eleftheria Zeggini.

The members of the ARGO-Larissa Consortium are Konstantinos Malizos, Lydia Anastasopoulou, Aspasia Tsezou, Eleni Zengini, J. Mark Wilkinson, Konstantinos Hatzikotoulas, and Eleftheria Zeggini.

The members of the Regeneron Genetics Center Consortium are Goncalo Abecasis, Aris Baras, Michael Cantor, Giovanni Coppola, Andrew Deubler, Aris Economides, Luca A. Lotta, John D. Overton, Jeffrey G. Reid, Alan Shuldiner, Katia Karalis, Katherine Siminovitch, Christina Beechert, Caitlin Forsythe, Erin D. Fuller, Zhenhua Gu, Michael Lattari, Alexander Lopez, Thomas D. Schleicher, Maria Sotiropoulos Padilla, Louis Widom, Sarah E. Wolf, Manasi Pradhan, Kia Manoochehri, Xiaodong Bai, Suganthi Balasubramanian, Boris Boutkov, Gisu Eom, Lukas Habegger, Alicia Hawes, Olga Krasheninina, Rouel Lanche, Adam J. Mansfield, Evan K. Maxwell, Mona Nafde, Sean O’Keeffe, Max Orelus, Razvan Panea, Tommy Polanco, Ayesha Rasool, William Salerno, Jeffrey C. Staples, Dadong Li, Deepika Sharma, Ilanjana Banerjee, Jonas Bovijn, Adam Locke, Niek Verweij, Mary Haas, George Hindy, Tanima De, Parsa Akbari, Olukayode Sosina, Manuel A. R. Ferreira, Marcus B. Jones, Jason Mighty, Michelle G. LeBlanc, and Lyndon J. Mitnaul.

## Data Availability

The data from the genome-wide summary statistics for each meta-analysis generated during this study have been deposited at the ‘Downloads’ page of the Musculoskeletal Knowledge Portal (https://mskkp.org), and are publicly available as of the date of publication. Details for the cohort-specific raw genetic/genomic data are listed in the key resources table. This paper does not report original code. The scripts used to run publicly available software (listed in the Key resources table) has been deposited at https://github.com/hmgu-itg/Genetics-of-Osteoarthritis-1 and is publicly available. The DOI is listed in the Key Resources Table. Any additional information required to reanalyse the data reported in this paper is available from the lead contact upon request.

## Supplemental information

Table S1. Phenotype definition and cohort information, related to Figure 1 and Genetic architecture: Identification of osteoarthritis risk variants GO cohorts included in the meta-analysis summarizing sample numbers, characteristics and analysis details.

Table S2. Summary statistics in GO meta-analysis for the previously reported osteoarthritis SNVs, related to Genetic architecture: Identification of osteoarthritis risk variants and STAR Methods

Table S3. Complete list of risk SNVs for 100 signals at genome-wide significance (p < 1.3 × 10^−8^) and their summary statistics in all osteoarthritis phenotypes, related to Table 2, Genetic architecture: Identification of osteoarthritis risk variants and Early-onset osteoarthritis, and Cross-phenotype analysis: Similarities and differences of signals across phenotypes

Table S4. PRS in 11 osteoarthritis phenotypes, related to Genetic architecture: Identification of osteoarthritis risk variants and STAR Methods

Table S5. Summary statistics for the sex-specific GO meta-analysis, showing SNVs with significant gender heterogeneity or gender-differentiated p value, related to Genetic architecture: Female-specific osteoarthritis risk variants and STAR Methods

Table S6. Genetic correlation matrix between the 11 GO osteoarthritis phenotypes and causal links with other traits and comorbidities, related to Figure 2E and Cross-phenotype analysis: Genetic links between phenotypes

Table S7. Plasma pQTL Mendelian randomization and colocalization results, related to Effector genes and biological pathways: Identification of putative causal variants and Amassing evidence to identify effector genes and STAR Methods

Table S8. Fine-mapping and GTEx colocalization results, related to Effector genes and biological pathways: Identification of putative causal variants and Amassing evidence to identify effector genes and STAR Methods

Table S9. In silico functional genomics lookup for all genes within 1 Mb of the 100 GO SNVs, related to Table 3 and Effector genes and biological pathways: Amassing evidence to identify effector genes

Table S10. Identification of putative causal genes through fine-mapping and functional genomics, related to Table 3 and Effector genes and biological pathways: Amassing evidence to identify effector genes

Table S11. Phenome-wide analysis of the associated variants to identify enrichment of signal for related traits and diseases, related to Effector genes and biological pathways: Amassing evidence to identify effector genes and STAR Methods

Table S12. Additional analyses to glean further insight into the biological role of the high-confidence effector genes in osteoarthritis disease processes, related to Figure 3 and Effector genes and biological pathways: Amassing evidence to identify effector genes

Table S13. Pathway analysis, related to STAR Methods and Effector genes and biological pathways Results of the three stratified pathway analyses: i) 637 genes with at least one line of evidence of causality ii) 77 high-confidence (at least three lines of evidence) and iii) Weight bearing/non-weight bearing
