# Deciphering osteoarthritis genetics across 826,690 individuals from 9 populations

**DOI:** 10.1016/j.cell.2021.11.003

**Published:** 2021-11-24

**Authors:** Cindy G. Boer, Konstantinos Hatzikotoulas, Lorraine Southam, Lilja Stefánsdóttir, Yanfei Zhang, Rodrigo Coutinho de Almeida, Tian T. Wu, Jie Zheng, April Hartley, Maris Teder-Laving, Anne Heidi Skogholt, Chikashi Terao, Eleni Zengini, George Alexiadis, Andrei Barysenka, Gyda Bjornsdottir, Maiken E. Gabrielsen, Arthur Gilly, Thorvaldur Ingvarsson, Marianne B. Johnsen, Helgi Jonsson, Margreet Kloppenburg, Almut Luetge, Sigrun H. Lund, Reedik Mägi, Massimo Mangino, Rob R.G.H.H. Nelissen, Manu Shivakumar, Julia Steinberg, Hiroshi Takuwa, Laurent F. Thomas, Margo Tuerlings, arcOGEN Consortium, HUNT All-In Pain, ARGO Consortium, Regeneron Genetics Center, George C. Babis, Jason Pui Yin Cheung, Jae Hee Kang, Peter Kraft, Steven A. Lietman, Dino Samartzis, P. Eline Slagboom, Kari Stefansson, Unnur Thorsteinsdottir, Jonathan H. Tobias, André G. Uitterlinden, Bendik Winsvold, John-Anker Zwart, George Davey Smith, Pak Chung Sham, Gudmar Thorleifsson, Tom R. Gaunt, Andrew P. Morris, Ana M. Valdes, Aspasia Tsezou, Kathryn S.E. Cheah, Shiro Ikegawa, Kristian Hveem, Tõnu Esko, J. Mark Wilkinson, Ingrid Meulenbelt, Ming Ta Michael Lee, Joyce B.J. van Meurs, Unnur Styrkársdóttir, Eleftheria Zeggini

(Cell *184*, 4784–4818.e1–e16, September 2, 2021)

In this article, we carried out a multi-cohort GWAS meta-analysis for 11 osteoarthritis phenotypes. Since publication, we have become aware of the following typographical errors that were introduced during preparation of the manuscript and resulted from multiple authors editing a single shared document.

In Table 1, there were two typographical errors: for spine osteoarthritis, the correct number of cases and controls is 28,372/305,578 (originally written as 28,3721/3057,578).

In Table 2, there were 11 typographical errors: HipOA rs781661531 EAF is 0.9997 (not 7 × 10^−4^), HandOA rs10062749 p is 2.04 × 10^−9^ (not 2.04 × 10^−09^), TJR rs116934101 OR is 1.06 (not 1.106), TJR rs10824456 OR is 0.95 (not 10.95), KneeOA rs1426371 OR is 0.95 (not 10.95), KneeHipOA rs551471509 EAF is 0.9996 (not 6 × 10^−4^), KneeOA rs11705555 p is 2.99 × 10^−9^ (not 3.00 × 10^−9^), KneeHipOA rs2856821 OR is 1.05 (not 1.11), female-specific AllOA rs10453201 95%CI is 1.03–1.06 (not 1.02–1.06), the nearest gene to female-specific THR rs116112221 is *FANCL* (not *FALCL1*), and the nearest gene to female-specific THR rs10282983 is *C8orf34* (not *C3ORF34*).

In Table S2, there were two typographical errors: cell 42E is 0.3 (not 0.72) and cell 42F is 1.06 (not 1.08).

In the discussion, fibrillin 2 was inadvertently labelled as *FNB2* in two places, instead of *FBN2*.

Finally, we inadvertently used an incorrect version of Figure 1, in which the information on the list of SNVs in (C) was incomplete and 12 typographical errors were introduced during the submission process. Specifically, in Figure 1A, the number of cases and controls of (1) Knee and/or Hip OA is 89,741 cases/ 400,604 controls (not 90,865 cases/ 402,824 controls), (2) Hand OA is 20,901 cases/ 282,881 controls (not 21,186 cases/ 285,101 controls), (3) Spine OA is 28,372 / 305,578 (not 28,731/307,798), (4) Hip OA is 36,445 cases/ 316,943 controls (not 36,520 cases/ 317,590 controls), and (5) Knee OA is 62,497 cases/ 333,557 controls (not 63,498 cases/ 335,777 controls). In Figure 1C, we have updated the SNVs listed in the panels, and the following sequences are now included: rs13107325, rs17615906, and rs3884606 in the Hip Osteoarthritis (and/or THR) section; rs11164653 in the Knee and/or Hip Osteoarthritis (and/or TJR) section; rs10062749, rs11071366, rs1530586, rs216175, rs58973023, and rs74676797 in the Knee Osteoarthritis (and/or TKR) section; rs11164653, rs1530586, rs17615906, and rs9908159 in the All OA section; and rs10062749 in the Hand and Knee Osteoarthritis or TKR section. Also, rs3771501 has been removed from the Hand and Knee Osteoarthritis or TKR section.

These errors have now been corrected in the online version of the paper. The authors apologize for any inconvenience they may have caused the readers.

**Figure 1:**
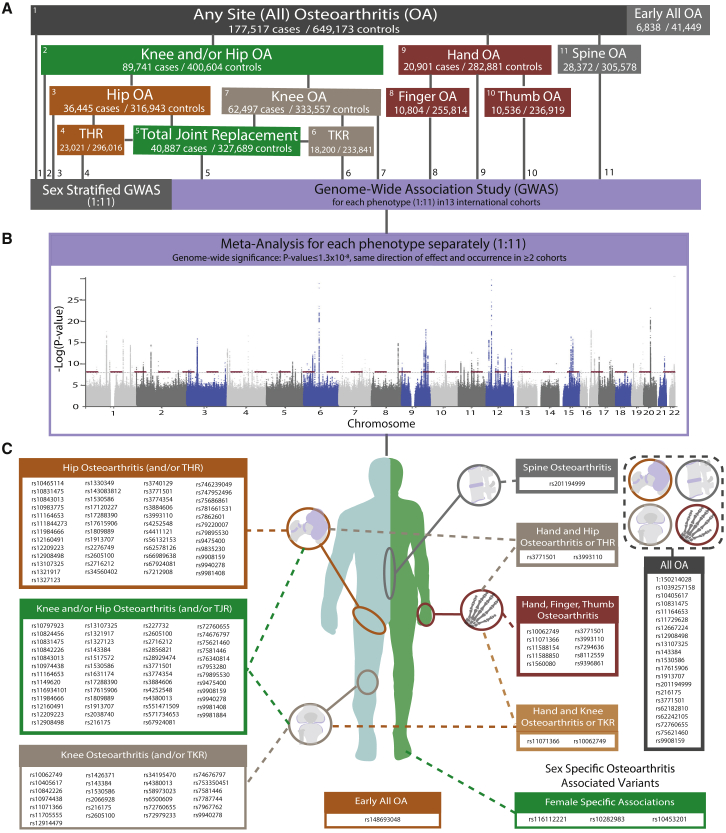
Genetic architecture (corrected)

**Figure 1:**
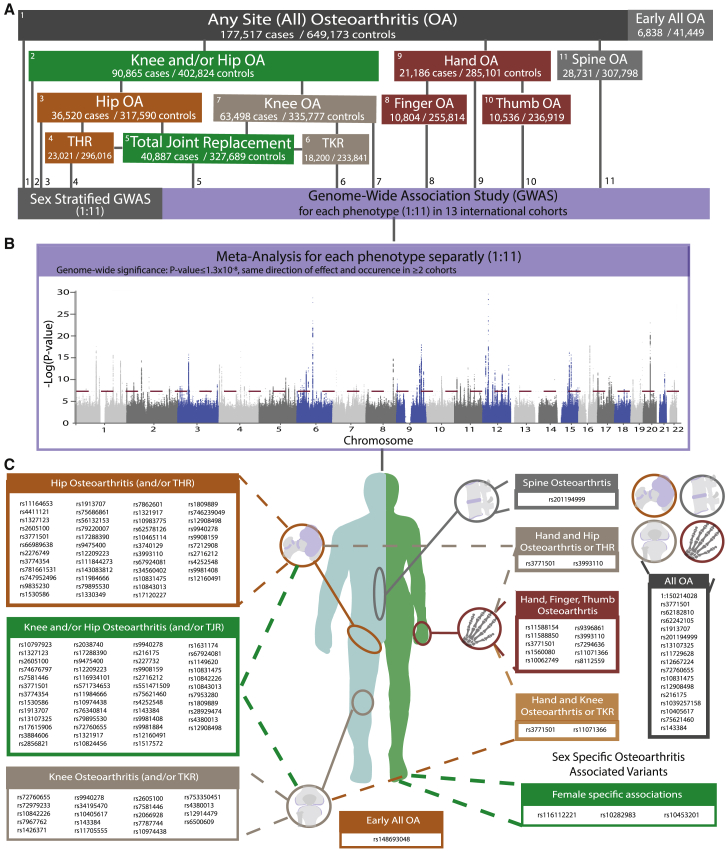
Genetic architecture (original)

